# A bacteria-based search for drugs against avian and swine flu yields a potent and resistance-resilient channel blocker

**DOI:** 10.1073/pnas.2502240122

**Published:** 2025-08-01

**Authors:** Hiya Lahiri, Eitan Israeli, Miriam Krugliak, Kingshuk Basu, Yelena Britan-Rosich, Tamar Ravins Yaish, Isaiah T. Arkin

**Affiliations:** ^a^Department of Biological Chemistry, The Alexander Silberman Institute of Life Sciences, The Hebrew University of Jerusalem, Jerusalem 9190400, Israel; ^b^Barry Skolnick Biosafety Level 3 Unit, The Hebrew University of Jerusalem, Jerusalem 9112102, Israel; ^c^Authority For Biological and Biomedical Models, The Hebrew University of Jerusalem, Jerusalem 9112102, Israel; ^d^Department of Biomedical Engineering, College of Biomedicine, City University of Hong Kong, Kowloon, Hong Kong Special, Administrative Regions of China

**Keywords:** influenza, channel blockers, ion channel

## Abstract

Influenza represents a significant threat with seasonal epidemics that can transition to global pandemics, and cross-species infection presenting a continuous challenge. While vaccines and several antiviral drugs are available, constant genetic changes vitiate these prevention and treatment options. Consequently, we decided to search for inhibitors against one of the virus’s validated drug targets, its M2 channel that is blocked by aminoadamantanes. Regrettably, widespread mutations in M2 abolish the antiflu activity of said blockers. Therefore, we devised bacteria-based genetic assays that can screen for drugs against aminoadamantane-sensitive and resistant M2 channels and map the resistance potential of any identifiable blocker. Subsequent in cellulo testing and structure–activity relationship studies yielded a synergistic combination of two compounds, Theobromine and Arainosine, that exhibited remarkable antiviral activity by directly inhibiting the virus’s channel. The drug duo was potent against H1N1 pandemic swine flu, H5N1 pandemic avian flu, and aminoadamantane-resistant and sensitive strains alike, exhibiting activity that surpassed oseltamivir, the leading antiflu drug on the market. When this drug duo was tested in an animal model, it once more outperformed oseltamivir, considerably reducing disease symptoms and viral RNA progeny. Importantly, harnessing the bacterial genetic selection, we could demonstrate that the drug duo’s potential for eliciting drug resistance is significantly smaller and molecularly distinct from that of aminoadamantanes. In conclusion, the outcome of this study represents a new potential treatment option for influenza alongside an approach that is sufficiently general and readily applicable to other viral targets.

## Significance

Influenza is an important health threat and a leading cause of mortality from an infectious disease globally. Vaccinations offer limited protection, and resistance emerged against all antiviral drugs. Therefore, the discovery of new antivirals is key. The current study aims to discover antivirals by targeting the pathogen’s channel. Using a combined approach, a synergistic combination of arainosine and theobromine is shown to be particularly potent against swine flu, avian flu, aminoadamantane-sensitive and resistant strains, outperforming oseltamivir, the leading antiflu drug. Additionally, the drug duo elicited less resistance in comparison to aminoadamantanes. The outcome of the study represents a promising antiviral candidate and an approach that can be applied to many other viruses.

Influenza presents a worldwide health challenge. Global estimates by the World Health Organization place the number of severe infections at 3 to 5 million, which lead to 290,000 to 650,000 deaths annually (1, 2). In particular, in the Western world before the COVID-19 pandemic, it was the leading cause of mortality from infectious diseases (3).

The etiological agent of influenza is the eponymous influenza virus, identified by Shoppe in 1931 as the cause of swine flu (4–7) and by Smith, Andrewes, and Laidlaw in 1933 as the agent behind the human disease (8). As a member of the *Orthomyxoviridae*, the virus is a negative-sense, single-stranded, segmented RNA virus. The family contains several genera, of which only the *Alphainfluenzavirus* and *Betainfluenzavirus* have members that pose a health risk. Since only the influenza A virus has been known to cause pandemics, it is the focal point of this study.

There are several prevention and treatment options for influenza: Annual vaccinations have been available for some time, but their efficacy is limited between 40% and 60% (1) due to the low fidelity of its RNA polymerase that causes a constant drift and shift in the virus’s genome. The principal antigens are the hemagglutinin and neuraminidase glycoproteins, of which there are 16 and 9 serotypes, respectively [not including bat influenza A-like viruses (9)]. Consequently, their combination is used as a primary descriptor of the strain, and in the current study, we focus on the two most significant health threats: H1N1 swine flu and H5N1 avian flu.

Antiviral agents are also available against the influenza virus, targeting several of the pathogen’s proteins (for a recent review, see ref. 10). The most common agents in current use are neuraminidase inhibitors such as oseltamivir, zanamivir, peramivir, and laninamivir. More recently, inhibitors were approved against the viral RNA polymerase and its complex (favipiravir and baloxavir) and the pathogen’s fusion machinery (umifenovir). However, the first anti-influenza agents were the aminoadamantanes, identified in 1964 (11). Mutations in M2 conferred resistance to said drugs, singling the protein as the target within the virus (12). In 1992, Lamb and colleagues, in a landmark study, showed that M2 is a H^+^ channel that is blocked by aminoadamantanes, thereby providing a molecular mechanism of their activity (13). Regrettably, resistance has developed against all aforementioned drugs (10) and is particularly prevalent against aminoadamantanes, rendering their utility obsolete (14).

The discovery of an ion channel coded by the influenza virus led to the identification of many such proteins, collectively termed viroporins (15). They are often characterized by their small size and oligomeric nature, with varying roles in the viral infectivity cycle. In influenza, M2 was shown to be essential to the pathogen since it allowed the concomitant acidification of the viral lumen upon endocytosis and release of the viral ribonucleoprotein from the matrix (13).

Considering the fact that channels, as a family, are excellent drug targets, and there is a dearth of curative anti-influenza agents, we sought to identify novel blockers against the M2 protein. In particular, we searched for blockers that will be active against aminoadamantane-sensitive and aminoadamantane-resistant channels. To that end, we employed a combined approach of bacteria-based assays, in cellulo assays, and structure–activity analyses that led to successful animal experimentation. Importantly, bacteria-based assays harness the power of bacterial genetics to examine the detailed interactions between a channel and its cognate blocker. Furthermore, such assays enable entirely risk-free generation of gain-of-function mutations and, combined with genetic selections, allow for the analysis of resistance probability against an antiviral drug. Together, this approach yielded a potent anti-influenza agent that is less likely to elicit viral resistance and represents a general route applicable to many other viruses.

## Results

### Channel Activity in Bacteria.

The channel activity of M2 was examined in bacteria, whereby conductivity that results from the protein changes the host’s phenotype. In brief, three bacteria-based assays–negative, positive, and pHlux assays (16–18)–were used to ensure reliability and minimize false results. This approach has been used successfully on multiple viral ion channels since their small size facilitates functional expression in bacteria (16–25). Moreover, this approach harnesses the power of bacterial genetics and facilitates genetic selections as expounded below.

In the negative assay, increased protein expression upon raising Isopropyl-*β*- D-1-thiogalactopyranoside (IPTG) levels causes retardation of bacterial growth due to excessive membrane permeabilization (16). For example, at an IPTG concentration of 45 μM, the bacterial growth is roughly halved compared to bacterial cells without an inducer (*SI Appendix*, Fig. S2*A*).

In the positive assay, which is reciprocal to the negative assay, K^+^-uptake-deficient bacteria are used since they can only grow in a high potassium medium (26). However, these bacteria can survive in low K^+^ medium when they express a channel capable of K^+^ transport, which leads to renewed bacterial growth. *SI Appendix*, Fig. S2*B* shows that appreciable bacterial growth is observed upon induction of protein expression. Note that lower IPTG concentrations are used in this assay since excessive permeabilization ensues at higher protein expression levels, and the benefit of potassium transport is nullified (17).

The final test is the pHlux assay (18), where injection of acid into the media results in a protein-mediated H^+^ influx into the bacteria that is measured by a pH-sensitive green fluorescent protein (GFP) (27). Different protein expression levels that result from different IPTG concentrations will yield different acidification rates. As seen in *SI Appendix*, Fig. S2*C*, significant acidification is observed when the inducer concentration increases to 50 μM. However, lower acidification is observed at inducer concentrations beyond 50 μM, most likely due to the detrimental impact of the protein on bacterial growth, as observed in the negative assay (*SI Appendix*, Fig. S2*A*).

In conclusion, all three assays indicate appreciable conductivity by the viral protein, manifested by a change in bacterial phenotype. Consequently, reversing the phenotype can be used in drug screening, as elaborated below.

### Drug Screening.

We employed the above bacteria-based assays to search for compounds that can inhibit (i.e., block) the activity of the influenza M2 channel. In particular, we used the negative assay as the primary screening tool since, in this instance, successful blockers will be identified due to their ability to enhance bacterial growth. Hence, the negative assay presents a stringent test requiring bacteria to grow faster than otherwise, thereby filtering out any compounds that are toxic to the host. In contrast, in the positive assay, successful compounds will be deleterious to growth, and in the pHlux assay, they will abrogate fluorescence change. Rimantadine was used as a positive control for all assays, while growth without drugs served as the negative control. In each of the three assays, an optimal protein expression level was selected to maximize the dynamic range of blocker activity. Finally, we employed a repurposed drug library numbering 2,839 compounds. Such a library represents a set of compounds with known toxicity and can be used as a starting point for further chemical exploration and subsequent examination of antiviral effect in mammalian cells and in vivo experiments.

**Fig. 1.**
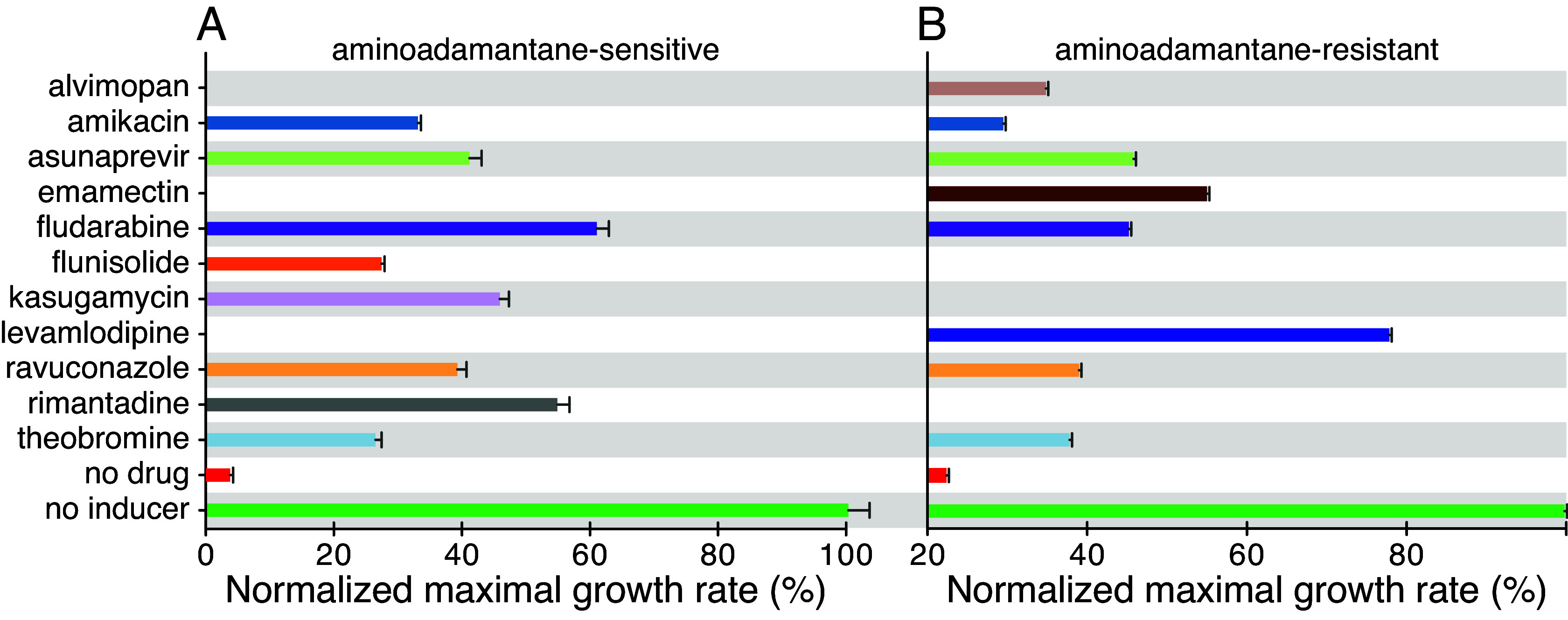
Screening results employing the negative assay against the aminoadamantane-sensitive M2 channel (*A*) and against a channel containing the S31N mutation (*B*) that renders the channel aminoadamantane resistant (28). Bacteria without the IPTG inducer (green) and untreated bacteria (red) serve as controls. Effective blockers increase bacterial growth. Note that rimantadine exhibits potency against the aminoadamantane-sensitive channel (*A*) but, as expected, has no activity against a channel with the S31N mutation and is incapable of restoring bacterial growth (*B*).

The above process led to seven compounds designated as hits against the aminoadamantane-sensitive channel (Fig. 1*A*): amikacin, asunaprevir, fludarabine, flunisolide, kasugamycin, ravuconazole, rimantadine, and theobromine. The same screening process was then employed on the M2 channel with the S31N aminoadamantane-resistant mutation (28) yielding eight hits, of which five were active against the aminoadamantane-sensitive channel as well (Fig. 1*B*): alvimopan, amikacin, asunaprevir, emamectin, fludarabine, levamlodipine, ravuconazole, and theobromine. As expected, rimantadine was a potent blocker of the aminoadamantane-sensitive channel, but was ineffective against the channel with the S31N mutation that is known to be aminoadamantane-resistant (28). Finally, all hits were also examined against their respective channel using the positive and pHlux assays yielding varying activities (*SI Appendix*, Fig. S3).

#### In Cellulo Antiviral Activity.

We proceeded to examine the antiviral activity of the blockers we identified in the bacteria-based assays. To that end, the pandemic H1N1/09 swine flu strain was selected since it was the first virus designated by the World Health Organization as a public health emergency of international concern (29). In addition, the H1N1/09 virus contains the S31N mutation circulating in the vast majority of current viruses (30), rendering them aminoadamantane resistant (28). The assay was based on the ability of the hit compounds to reduce viral-induced cellular death in tissue culture. Oseltamivir, a neuraminidase inhibitor (31) and uninfected cells served as positive controls, while untreated cells as negative controls.

#### Compound toxicity assessment.

Prior to efficacy analysis, it was imperative to determine the toxicity of the different hits. Hence, a tolerability test was performed to confirm the dose range up to which the hit concentration is safe to use in the efficacy studies. Specifically, we tested the cellular toxicity of each compound at 1 to 30 μM, by monitoring the cell viability after 48 h. As shown in *SI Appendix*, Fig. S4, all compounds do not exhibit toxicity up to 30 μM, with the only exception of levamlodipine, that exhibits increasing toxicity from 3 μM upward.

#### Antiviral efficacy of hit compounds.

Having examined the toxicity of the different hits, we could examine their antiviral activity in cellulo. With an A/Wisconsin/629-D02452/2009 (H1N1)pdm09 virus with an S31N M2 channel, we tested the ability of compounds to reduce viral-induced death. The compounds were tested at 10 μM (or lower if toxicity studies did not enable such a high concentration), and the viability of the cells was examined two days post infection.

The results shown in *SI Appendix*, Fig. S5 demonstrate that at a multiplicity of infection (MOI) of 0.3, more than 55% of the cells are nonviable after two days due to viral infection. However, several of the hits obtained from the bacterial screening were found to increase cell viability considerably. In particular, asunaprevir, fludarabine, flunisolide, levamlodipine, and theobromine exhibited appreciable activity in negating virus-induced cellular death. Oseltamivir, serving as a positive control can almost entirely prevent viral-induced death. Finally, as expected, rimantadine exhibited no activity whatsoever, since the viral strain is aminoadamantane-resistant.

#### Activity of structurally similar compounds.

All five compounds that exhibited appreciable in cellulo activity at 10 μM were subjected to a search for structurally similar compounds. In doing so, we could fully utilize the repurposed drug library and uncover additional compounds that may not have scored positively in the bacteria-based assay but could still possess antiviral activity.

A search for compounds similar to fludarabine yielded vidarabine, while theobromine yielded theophylline, and asunaprevir yielded grazoprevir. Other search outcomes yielded compounds that did not exhibit any antiviral activity. We then conducted a dose–response analysis for all the above-mentioned active compounds, including both the parent and similar compounds. Two commercially available anti-influenza drugs, oseltamivir and favipiravir (viral RNA-dependent RNA polymerase inhibitor) were used as positive controls.

**Fig. 2.**
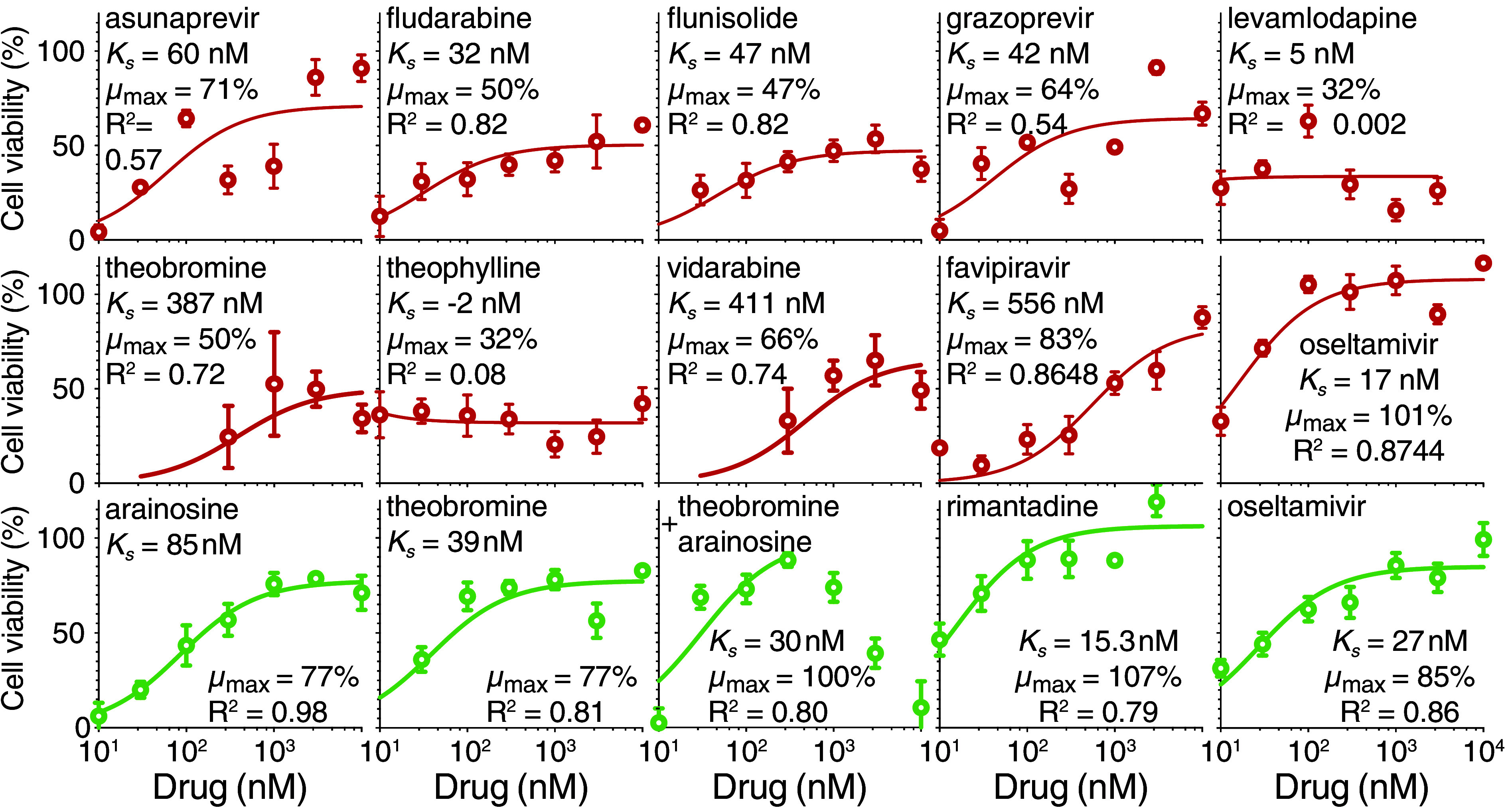
In cellulo antiviral dose–response studies of active drugs. MDCK cells were infected with an H1N1 aminoadamantane-resistant (in red) or H5N1 aminoadamantane-sensitive (in green) virus at an MOI of 0.3 and their viability was monitored by MTS after 48 h. Results are normalized relative to uninfected cells and untreated cells. *K_s_*, $\mu_{\text{max}}$, and corresponding R^2^ values are shown for each drug in the *Inset*. Note that for the theobromine and arainosine drug combination, equal concentrations were used and the fit employed data until 0.3 μM.

Results shown in Fig. 2 indicate that the following compounds exhibit activity with a well-defined dose–response characteristic: asunaprevir (*K_s_* = 60 nM, $\mu_{\text{max}}$ = 71%), fludarabine (*K_s_* = 32 nM, $\mu_{\text{max}}$ = 50%), flunisolide (*K_s_* = 47 nM, $\mu_{\text{max}}$= 47%), grazoprevir (*K_s_* = 41 nM, $\mu_{\text{max}}$ = 64%), theobromine (*K_s_* = 387 nM, $\mu_{\text{max}}$ = 50%), and vidarabine (*K_s_* = 411 nM, $\mu_{\text{max}}$ = 66%). Yet, individually, none of the compounds was efficacious as oseltamivir (*K_s_* = 17 nM, $\mu_{\text{max}}$ = 101%).

#### Drug combination studies.

**Fig. 3.**
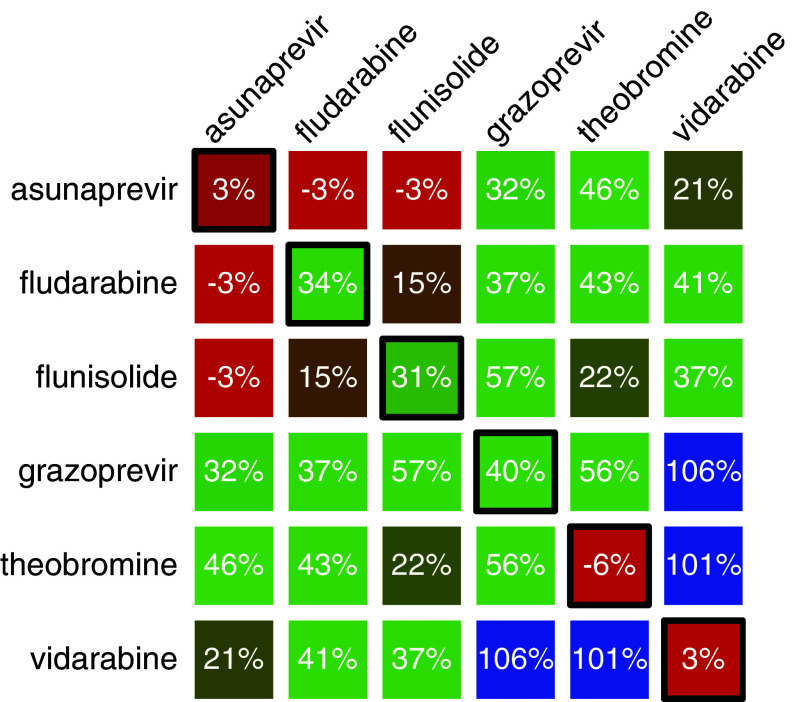
Combination antiviral in cellulo studies of asunaprevir, fludarabine, flunisolide grazoprevir, theobromine, and vidarabine. MDCK cells were infected with the H1N1 virus at an MOI of 0.3 and their viability was monitored by MTS after 48 h. All compounds were at 0.1 μM. Diagonal elements (boxed) represent activity of the individual drugs. Results are normalized relative to uninfected cells and untreated cells and represent the average of at least three measurements.

To examine any beneficial antiviral activity of multiple drugs we studied the effect of drug combinations at 0.1 μM starting point. As shown in Fig. 3, few combinations showed pronounced synergism compared to their individual components. In particular, the combination of theobromine and vidarabine was able to completely protect cells from virus-induced cellular death, while individually, no activity at that concentration was observed. The combination of vidarabine with grazoprevir was also potent, but in this instance, grazoprevir on its own restores viability to 56% compared to untreated cells. All other combinations exhibited additivity, or no added effect.

Having identified a remarkable synergism between theobromine and vidarabine, we sought to characterize it further. In *SI Appendix*, Fig. S6, we present the results of a combination study at concentrations from 10 to 300 nM. Once again, stark synergism is observed, but this time even at lower concentrations, whereby at an equal molar ratio of 30 nM, near complete protection from virus-induced cellular death is observed. For comparison, two approved anti-influenza drugs at 30 nM, oseltamivir and favipiravir, provide only 71% and 10% protection, respectively.

Interestingly, antiviral synergism seems to decrease at higher concentrations. For example, at an equal ratio of 30 nM, the drug duo exhibits 97% protection but the activity wanes to 83% and 3% at 100 nM and 300 nM, respectively.

Metabolically, vidarabine rapidly deaminates to arainosine by adenosine deaminase (32). Therefore, we decided to examine the activity of arainosine, individually and in combination with theobromine. As seen in *SI Appendix*, Fig. S6, individually, arainosine is perhaps slightly more effective than vidarabine, but both compounds exhibit ca. 34% activity at 0.3 μM, whereas oseltamivir is 100% effective at that concentration. However, once again, dramatic synergism is observed, where complete protection from virus-induced death is observed at 10 nM of arainosine with 30 nM of theobromine. These results indicate that the active form of vidarabine is its deaminated metabolite–arainosine. Finally, a decrease in activity at higher concentrations is observed once again, but to a lesser extent.

#### Activity against aminoadamantane-sensitive virus.

So far, the remarkable synergism of theobromine and arainosine has been demonstrated against an aminoadamantane-resistant viral strain. Therefore, we repeated the experiments on an H5N1, highly pathogenic avian influenza virus (Israel/975/2023) that, according to the sequence of its M2 channel, should be aminoadamantane-sensitive (*SI Appendix*, Fig. S1). Results shown in Fig. 2 indeed demonstrate that the virus is aminoadamantane-sensitive, whereby rimantadine is a potent inhibitor of infectivity, while against the H1N1 virus, it showed no activity whatsoever (*SI Appendix*, Fig. S5). Furthermore, the *K_s_* measured against the virus (15.3 nM) is indistinguishable from previous results (13 ± 4 nM) obtained in a bacteria-based assay (16).

Turning to the drug duo, both theobromine and arainosine individually exhibited activity against the aminoadamantane-sensitive avian influenza (Fig. 2). Yet, the activity of the individual compounds is significantly better against the aminoadamantane-sensitive strain relative to the activity against the aminoadamantane-resistant strain. For example, theobromine exhibited an affinity better by an order of magnitude alongside higher maximal activity. However, combining the two drugs against the aminoadamantane-sensitive strain leads only to mild additivity rather than stark synergism, which is observed against the aminoadamantane-resistant strain. Specifically, when both drugs are combined at 30 nM, similar protection from viral-induced death is observed against the aminoadamantane-sensitive and aminoadamantane-resistant strains (69% and 86%, respectively).

#### Inhibition site.

In order to ascertain the target of the theobromine and arainosine drug duo, we examined its binding properties to the M2 protein. Previously, we have shown that adamantanol is a competitive inhibitor of aminoadamantanes (33): It shares the adamantane moiety that enables it to bind the M2 channel but lacks a positive charge that repels H^+^ permeation. Consequently, we tested whether adamantanol can inhibit the activity of theobromine, arainosine, and their combination in the bacteria-based assay and in cellulo.

**Fig. 4.**
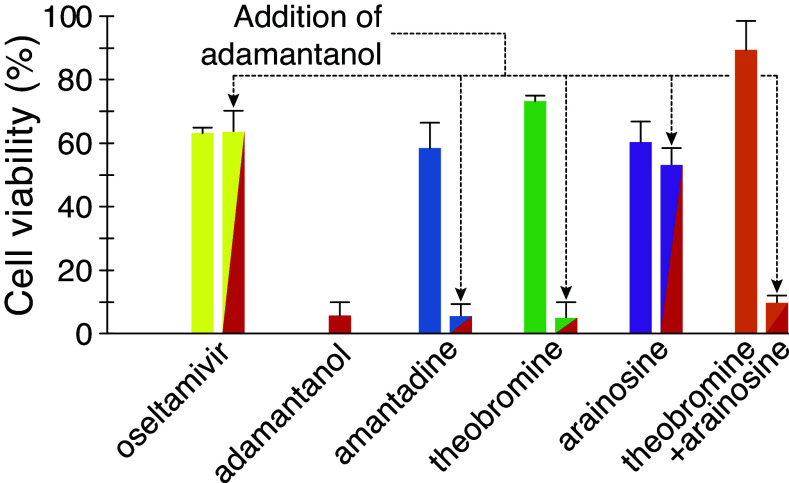
In cellulo binding mechanism analyses of theobromine and arainosine. MDCK cells were infected with the H5N1 virus at an MOI of 0.3, and their viability was monitored by MTS after 48 h. All drugs are given at 300 nM except adamantanol, which, on its own or in combination with other drugs, is administered at 5 μM. Results are normalized relative to uninfected cells and untreated cells.

As seen in Fig. 4, amantadine can effectively relieve viral-induced cellular death caused by the H5N1 virus. It does so by blocking the viral channel, which is essential to its infectivity cycle (13). However, addition of adamantanol (5 μM) abrogates the utility of amantadine almost entirely because it is its competitive inhibitor (33). Similarly, the activity of amantadine in the bacteria-based assays is nullified by adamantanol: In the negative assay, the ability of amantadine to relieve the growth inhibition due to excessive membrane permeabilization of the channel is negated by adamantanol (*SI Appendix*, Fig. S9*A*). In the pHlux assay, amantadine blocks the H^+^ flux through the channel, but adamantanol counteracts this activity (*SI Appendix*, Fig. S9*B*).

Repeating the above examination of adamantanol’s impact on the antiviral activity of theobromine, arainosine, and their combination reveals a similar picture (Fig. 4). Theobromine at 300 nM provides cells with 73% protection from viral-induced death. However, adding adamantanol reduces the protection to a mere 5%. In arainosine, a much smaller reduction is observed: The drug provides cells with 60% protection, which drops to 53% upon adding adamantanol. However, the activity of adamantanol is particularly pronounced in the theobromine-arainosine combination: the drug duo provides cells with 89% protection from viral-induced death that drops to 10% upon adamantanol addition. As expected, adamantanol has no protective effect against the virus, nor can it vitiate the benefit of oseltamivir, which targets the viral neuraminidase. Finally, these results are once more mirrored in the bacteria-based assays (*SI Appendix*, Fig. S9).

#### Structural activity relationship study.

Given the stark synergism between theobromine, a xanthine derivative, and vidarabine or arainosine, both nucleoside analogs (*SI Appendix*, Fig. S6), we sought to explore its specificity by examining highly similar analogs. In particular, we determined the antiviral activity of individual xanthine and nucleoside analogs and combinations thereof.

**Fig. 5.**
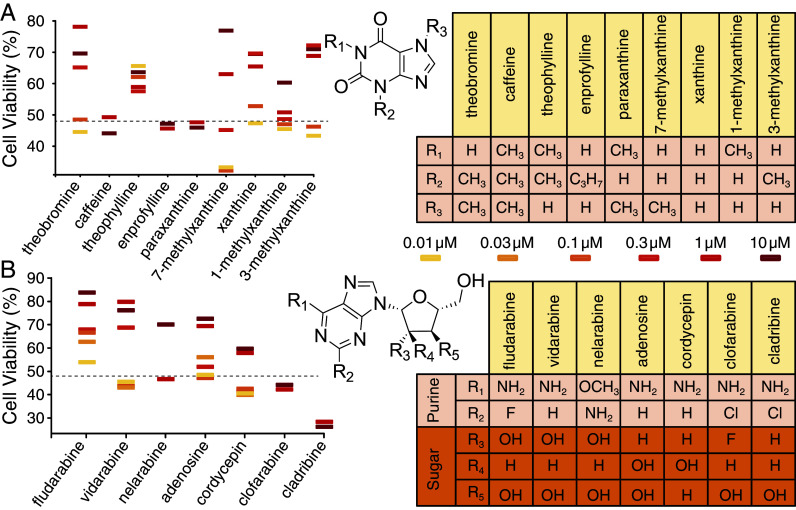
Structural activity relationship study of theobromine (*A*) and vidarabine (*B*). MDCK cells were infected with the H1N1 virus at an MOI of 0.3 and their viability was monitored by MTS after 48 h. Dashed lines indicate viability of untreated cells, while uninfected cells are set to 100%.

Theobromine is a xanthine-group member with multiple available structural analogs: caffeine, theophylline, enprofylline, paraxanthine, 7-methylxanthine, xanthine, 1-methylxanthine, and 3-methylxanthine (Fig. 5*A*). Among them, 7-methylxanthine shows higher activity than theobromine at 10 μM concentration. Apart from that, xanthine and 3-methylxanthine show comparable activity as theobromine at a higher concentration range (0.3 to 10 μM).

Vidarabine is a nucleoside analog of adenosine in which the ribose is epimerized to D-arabinose (34). Arainosine is the main metabolic byproduct of vidarabine and is obtained by deamination (32). Therefore, we sought to compare the antiviral activity of structurally similar analogs like fludarabine, nelarabine, cordycepin, clofarabine, cladribine, and adenosine.

Fig. 5*B* shows the activity of each drug at different concentrations along with the generalized structure of the nucleoside (*Inset* and table). Fludarabine exhibits the highest activity, followed by vidarabine, adenosine, and nelarabine. Other compounds have insignificant positive effects on cell viability.

We next proceeded to examine potential synergies between all of the nucleoside analogs and xanthine analogs. As seen in *SI Appendix*, Fig. S7, the only significant synergistic combination is between theobromine and vidarabine/arainosine. Interestingly, even though fludarabine was more potent than vidarabine/arainosine individually, it did not exhibit synergism in combination with theobromine (or any other xanthine derivative).

### In Vivo Study.

After demonstrating the synergism between theobromine and arainosine, we examined whether the drug duo exhibits antiviral activity in animals. Initially, we established the drug duo’s safety in mice. While the individual drugs are known to be very tolerable, the impact of their combination has not been established. In brief, no adverse effects were observed in all dosages tested, up to and including 100 mg/kg of theobromine with 150 mg/kg of arainosine (data not shown).

Subsequently, we infected mice with the influenza strain A/Wisconsin/629-D02452/2009 (H1N1)pdm09, and monitored the amount of viral RNA in the lungs after five days. During the course of infection, the mice were treated with the theobromine-arainosine drug duo at five different dosages alongside oseltamivir that served as a positive control, and a placebo that served as a negative control. All treatments were administered twice daily by oral gavage.

**Fig. 6.**
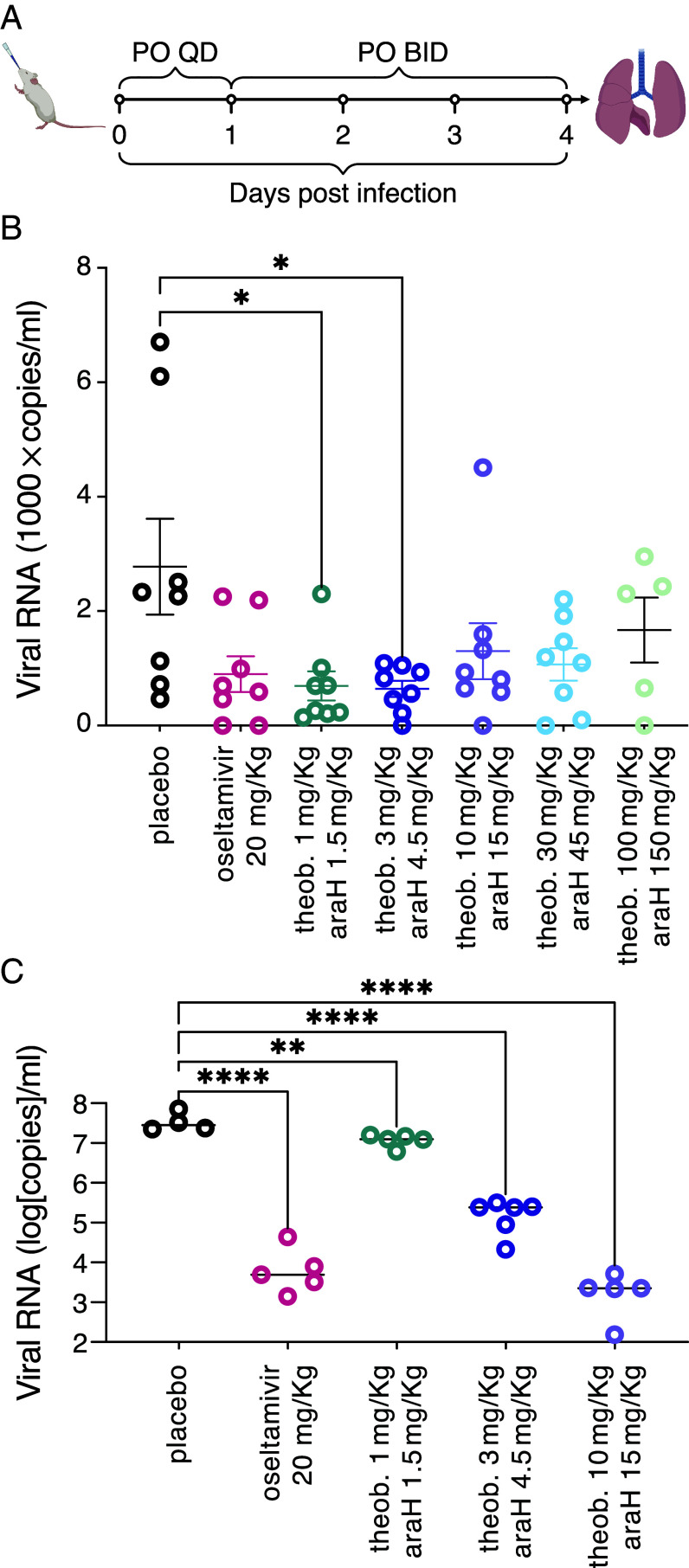
In vivo antiviral activity. (*A*) Scheme of experimental protocol. (*B*) Mice were infected with the A/Wisconsin/629-D02452/2009 (H1N1)pdm09 strain and viral RNA in the lung was measured after five days (*C*). Same as (*B*), but mice were infected with the PR8 H1N1 strain.

From the results shown in Fig. 6*B*, viral RNA load quantification showed a reduction compared to placebo of the following drug combinations: 1 mg/kg theobromine with 1.5 mg/kg arainosine and 3 mg/kg theobromine with 4.5 mg/kg arainosine (75% and 77%, respectively). Oseltamivir at 20 mg/kg and 10 mg/kg of theobromine with 15 mg/kg arainosine showed a reduction in viral load to a lesser extent which was not statistically significant relative to the placebo (57% and 53%, respectively). At higher concentrations of 30 and 100 mg/kg theobromine combined with 45 and 150 mg/kg arainosine, respectively, the percentage of viral load reduction decreased.

The above influenza strain does not elicit pronounced disease symptoms in the mice. Therefore, we transitioned to the mouse adopted PR8 H1N1 strain that is particularly virulent (35). Note that results in tissue culture demonstrate indistinguishable susceptibility of the PR8 strain to oseltamivir and the theobromine-arainosine duo (*SI Appendix*, Fig. S8). As seen in Fig. 6*C*, pronounced differences are observed between all treatments and the placebo. Oseltamivir at 20 mg/kg, reduces the viral load by 3,077 fold, while the combination of 10 mg/kg theobromine with 15 mg/kg arainosine leads to a reduction of 15,717 fold. Lower concentrations of theobromine with arainosine yield statistically significant reductions as well.

**Fig. 7.**
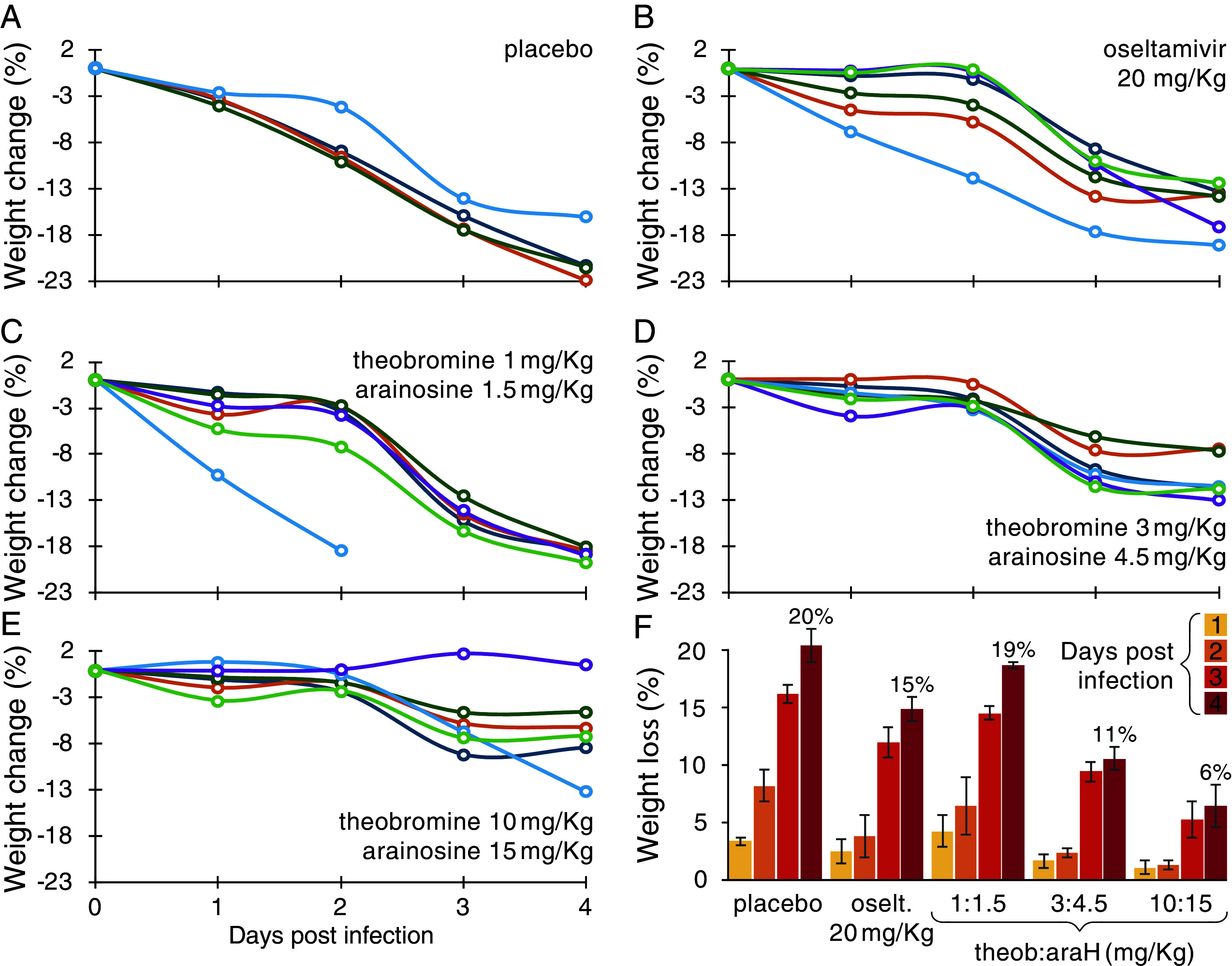
In vivo antiviral activity. Relative weight changes of mice receiving different treatments, as indicated, during the course of the infection. (*A*–*E*) Individual mice weight changes. (*F*) Average data for comparative purposes whereby values of four days post infection are listed in the graph.

Since mice infected with the PR8 strain exhibited pronounced disease symptoms, we could compare the effects of the different treatments thereupon. In particular, we monitored the mice’s weight as an indicative metric for disease progression. As shown in Fig. 7, mice that were treated with the placebo exhibited severe symptoms, losing ca. 20% of their body weight in five days. Oseltamivir at 20 mg/kg, as a positive control reduced the weight loss to less than 15%. However, the drug duo was significantly more effective once the dosage was raised to 3 mg/kg theobromine combined with 4.5 mg/kg arainosine. At a dosage of 10 mg/kg theobromine combined with 15 mg/kg arainosine the weight loss was reduced even further to only 6% after five days.

### Examination of Resistance Potential.

In order to analyze potential resistance against the drug duo, we exploited the bacteria-based genetic selection. In this construct, the protein can be exhaustively mutated to search for completely safe gain-of-function resistant mutations against any blocker. To that end, we constructed a library of bacteria, each harboring a different, randomly mutated M2 channel. The bacteria were then grown in liquid media, and next generation sequencing was employed after three hours to sequence the different channels. A comparison was conducted between bacteria grown without induction of protein expression, and in the presence of rimantadine or theobromine.

**Fig. 8.**
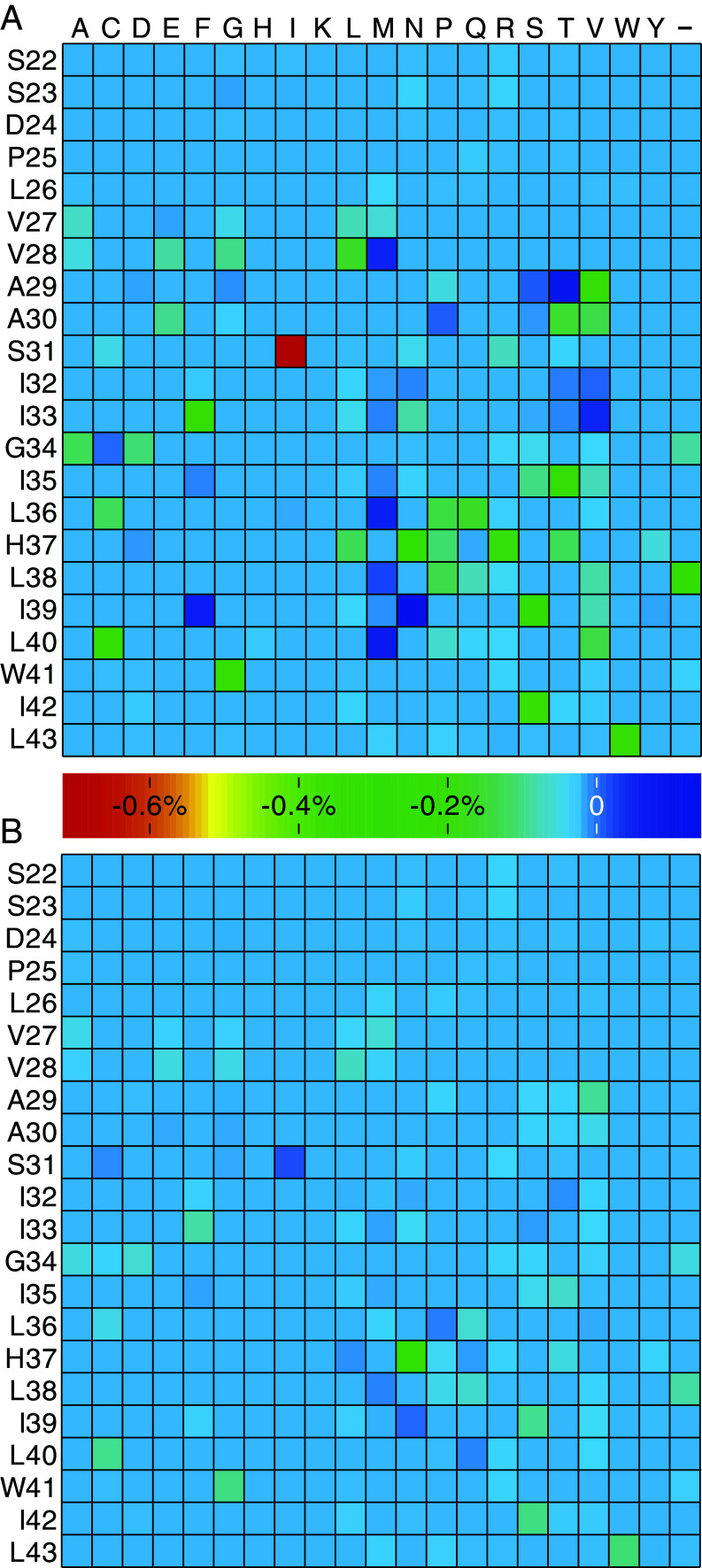
Analysis of the resistance potential against the M2 channel blockers. A library of bacteria harboring a randomly mutated M2 channel was grown in liquid media for three hours after which their plasmids were sequenced. The prevalence of every amino acid is tabulated and color-coded as indicated in the bar. The data correspond to difference prevalence of bacteria that are exposed to (*A*) rimantadine or (*B*) theobromine relative to untreated bacteria.

The results of roughly two million reads per treatment are shown in *SI Appendix*, Fig. S10. While differences between the different treatments are apparent, what is most informative are comparisons shown in Fig. 8. In these analyses, we compare the prevalence of every amino acid at every position in the protein when exposed to rimantadine or theobromine relative to untreated bacteria. For example, the mutation S31I is significantly disfavored when the bacteria are exposed to rimantadine. In contrast, bacteria that express a channel with the mutation I39N grow faster in the presence of rimantadine.

Interestingly, in contrast to rimantadine, growth in the presence theobromine does not appear to favor particular mutations appreciably. Moreover, the pattern of mutation prevalence is distinct between the two drugs. For example, bacteria that express a channel with the S31I mutation grow slower in the presence of rimantadine but faster in the presence of theobromine. In contrast, bacteria that express a channel with the I39F mutation grow faster in the presence of rimantadine but slightly slower in the presence of theobromine.

## Discussion

Influenza poses a severe health challenge, yet there is a dearth of curative antiflu agents. Furthermore, the virus’s constant genetic changes continuously vitiate even the partial benefit of current treatments. Consequently, we have searched for potential antiviral drugs for clinical application by attempting to identify new inhibitors of the virus’s viroporin.

Our approach, schematically shown in *SI Appendix*, Fig. S11, involved several steps: i) Identification of hits from a repurposed drug library with bacteria-based assays. ii) Addition of similar compounds. iii) Analysis of antiviral activity in tissue culture. iv) Combination studies to determine additivity and synergism. v) Efficacy determination in animals. Finally, we focused on the two most important strains that represent a pandemic threat: H1N1 swine flu and H5N1 avian flu. Moreover, we searched for agents that are active against both aminoadamantane-sensitive and resistant viral strains.

The focus of our study was to identify new anti-influenza agents by blocking its M2 channel, rather than detailed electrophysiological characterization. For this reason, (16–18, 33) we and others (36, 37) have shown that cell-based assays can effectively demonstrate the activity of blockers on the influenza M2 channel. We recognize that expression of heterologous proteins can potentially impact bacterial phenotype in many ways. Commensurately, the activity of inhibiting compounds may be ascribed to functionalities other than channel-blocking.

However, we demonstrate that it is M2’s channel activity that impacts the host’s phenotype, and the activity of the drugs on the host is due to their channel-blocking functionality as follows: i) We use three independent and complementary assays to measure channel activity (*SI Appendix*, Fig. S2). Moreover, two assays are reciprocal in nature, whereby in the first, channel activity retards bacterial growth, while in the second, channel activity enhances growth. ii) We demonstrate specificity by reversing the channel-driven phenotype in each of the different assays using aminoadamantanes, known blockers of the channel (11) (Fig. 1). iii) The phenotype of cells that express a channel that is resistant to aminoadamantanes cannot be reversed by blockers (Fig. 1). iv) Adamantanol, a competitive inhibitor of aminoadamantanes (33), can prevent the reversal of the channel-driven phenotype by blockers (*SI Appendix*, Fig. S9). v) The blockers are effective in multiple different hosts and systems: bacteria, tissue culture, and animals.

The outcome of the bacteria-based search yielded several blockers that encouragingly exhibited antiviral activity in tissue culture. Moreover, combination studies yielded a particularly synergistic drug duo of arainosine and theobromine that outperforms oseltamivir considerably (Fig. 3).

It is interesting to note that synergism is reduced at higher concentrations of both compounds. Mechanistically, Harris and coworkers have shown that sugars act as selective hydrotropes toward caffeine (38). Therefore, it is possible to speculate that the sugar moiety of vidarabine may similarly serve as a hydrotrope to theobromine, which differs from caffeine by a single methyl group. Finally, the combination of the two compounds exhibited striking activity in animals, that once more appreciably outperformed oseltamivir, the leading drug on the market against influenza (Figs. 6 and 7).

There is always a risk that compounds identified by their ability to inhibit a particular viral component in isolation inhibit the whole virus by an off-target route. In other words, while the compounds were selected based on their ability to block the M2 channel, they may exert their antiviral activity through different mechanisms. However, the fact that several structurally unrelated compounds retrieved from the bacteria-based selection for channel blockers exhibit antiviral activity suggests a low probability of an off-target effect.

It is possible to rule out an off-target mechanism altogether through the use of adamantanol, an antagonist of aminoadamantanes. The drug is an analog of amantadine but lacks a positive charge and, consequently, does not block the M2 channel (33). However, because it shares the adamantane group with aminoadamantanes, it can bind the channel and competitively inhibit their blocking function (33). The competitive inhibition of adamantanol can also be seen when analyzing activity against the entire pathogen in tissue culture where it abrogates the antiviral activity of amantadine almost entirely (Fig. 4). Encouragingly, adamantanol has the same neutralizing activity on theobromine, arainosine (to a lesser extent), and their combination (Fig. 4). Hence, it is challenging to conjure up a scenario in which the drugs inhibit the virus by any mechanism other than blocking its M2 channel. Finally, as expected, adamantanol has no antiviral effect on its own, nor does it impact the activity of oseltamivir since the drug works by an entirely different mechanism as a neuraminidase inhibitor (31).

It is also not possible to ascribe the remarkable anti-influenza activity of the drug duo based on the known anticancer and anti-DNA virus activity of vidarabine: Vidarabine deamination renders the drug inactive (39), while in the current study arainosine, the deaminated metabolite, is as effective, if not more, than vidarabine. This result is not surprising since the influenza virus does not go through a “DNA phase” during its infectivity cycle. Hence, the activity of arainosine on influenza should employ a different mechanism (i.e., blocking the M2 channel), as the impact of adamantanol suggests.

Importantly, the activity of the drug duo was effective against aminoadamantane-resistant and aminoadamantane-sensitive strains. Yet, the activity of the compounds, when used on their own, distinguishes the two strains. At 30 nM, no activity is observed for either drug against the aminoadamantane-resistant H1N1 strain (Fig. 2 and *SI Appendix*, Fig. S6). Yet, at the same concentration, both compounds exhibit appreciable but nonmaximal activity against the aminoadamantane-sensitive H5N1 strain (Fig. 2). Hence, either drug can inhibit (partially) the aminoadamantane-sensitive strain. At the same time, their combination is required for complete protection. In contrast, the individual drugs offer no benefit against the aminoadamantane-resistant variant, yet their combination provides full protection.

Further insight into the stark synergy between theobromine and arainosine in the aminoadamantane-resistant strain, was obtained by exploring the activity of similar compounds, individually (Fig. 5) and in combination (*SI Appendix*, Fig. S7).

Comparison of xanthine with 3- and 7-methylxanthine indicates that at higher concentrations, it is less active than both of its methylated derivatives. At lower concentrations, 3-methylxanthine is more efficacious than its 7-methyl analog. Yet, all three compounds are less effective than theobromine. Therefore, it can be concluded that methylations at the 3 and 7 positions are necessary to yield optimal antiviral effects.

1-methylxanthine, though, has an effect at a lower concentration range but does not show an effect at a higher concentration. On the other hand, theophylline, which is methylated at the 1 and 3 positions is active to some extent but shows poor dose-dependent activity (Fig. 2). Notably, the 1,7-dimethylated analog, paraxanthine, exhibits even lower activity. These observations further emphasize the role of methyl groups at the 3 and 7 positions, and any deviation from that leads to loss of activity. Moreover, the molecule completely loses all activity when all three nitrogen atoms are methylated, as in caffeine. Finally, to check the activity of the alkyl group, we employed enprofylline or 3-propylxanthine and found no effect when compared to its methyl analog 3-methylxanthine.

Arainosine or its metabolic precursor vidarabine are nucleoside analogs in which a purine is N-linked to a pentofuranose. While not as numerous as the analogs of theobromine, we examined several commercially available analogs for their antiviral activity in cell viability assays (Fig. 5*B*).

Fludarabine was the lead compound identified by the bacteria-based channel assays and in the in cellulo experiments. Therefore, compared to vidarabine, a Fluorine at R_2_ in the purine ring (the only point of difference with vidarabine) does not hamper the activity. On the other hand, substituting −NH_2_ at R_1_ with a −OCH_3_ group, as in nelarabine, significantly reduces activity (only active at 10 μM).

Analysis of the sugar moiety leads to a complex picture. Epimerization at the 2^′^ position, i.e., converting vidarabine to adenosine, increases the compound’s activity at lower concentrations but leads to a loss of activity at higher concentrations. Substitution of the sugar 3^′^−OH with a −H, found in cordycepin, also hampers activity. Interestingly, for clofarabine and cladribine, the scenario is entirely different: Inclusion of a different halogen atom (e.g., Cl) in the 2^′^ position of the purine ring with concomitant fluorination (in clofarabine) or reduction (in cladribine) at the sugar 2^′^ completely abolishes the antiviral activity. These observations illuminated the importance of purine ring −NH_2_ at R_1_ and sugar −OH at 2^′^ for the antiviral activity of the drugs. Finally, examining the 72 combinations of each of the nine vidarabine derivatives with each of the eight theobromine analogs indicates that the only potent combination is found between arainosine (or its prodrug vidarabine) and theobromine.

**Fig. 9.**
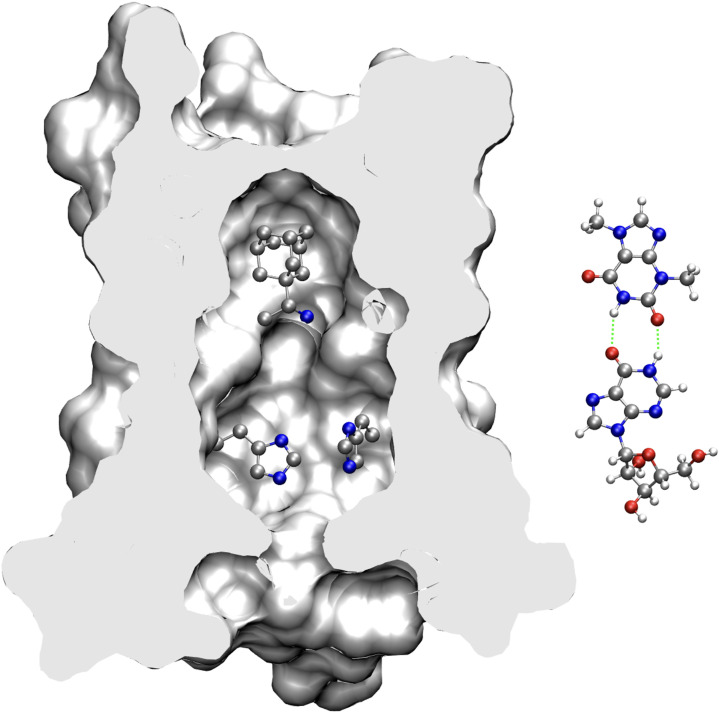
Proposed mechanism for inhibition of the arainosine-theobromine drug duo. The structure of the M2 protein bound to rimantadine [PDBID 6US9, (40)] is sliced in the *Middle* to depict the channel’s pore and drug binding site. Two out of the four histidines that are important for channel activity are depicted in ball-and-stick alongside rimantadine. Theobromine (*Top*) and arainosine (*Bottom*) are shown on the *Right* with proposed hydrogen bonding in green.

Clearly, detailed structural studies will be needed to explain the remarkable activity of theobromine with arainosine. However, based on the results presented in this study, it is possible to speculate as follows (Fig. 9): The activities of theobromine and aminoadamantanes are similarly abolished by adamantanol (Fig. 4), pointing to a shared binding site. Moreover, theobromine a planar molecule, is narrower than amantadine and, therefore, may still bind and inhibit the aminoadamantane-resistant S31N M2 channel, albeit at a substantially lower affinity than the aminoadamantane-sensitive channel. However, against aminoadamantane-sensitive strains, theobromine is individually less effective than aminoadamantanes, perhaps because its narrower dimension can only partially block proton transport. In contrast, adamantanol reduces the activity of arainosine only moderately, but abolishes the activity of the duo entirely. Hence, it is possible to speculate that arainosine’s binding site is located where the pore widens, and only partially overlaps with the binding site for aminoadamantanes.

Synergism between theobromine and arainosine may be explained based on their hydrogen bond compatibility (Fig. 9). In particular, caffeine that differs from theobromine by a methyl at the R1 position (Fig. 5*A*) exhibits no synergism with arainosine, consistent with the proposed H-bonding complementarity between the two compounds. There are other xanthine derivatives with a hydrogen at the R1 position (7-methylxanthine, xanthine, and 3-methylxanthine) and may be capable of such complementarity. However, their lack of synergism with arainosine may be due to the absence of an additional methyl group required for effective channel-blockage.

Efficacy is not the only attribute that is important to the utility of an antiviral drug. Equally as important is the potential of the virus, due to its rapid change, to develop resistance against the drug. The M2 channel is an infamous example of the above paradigm, whereby it developed widespread resistance against aminoadamantanes, essentially removing them from the list of viable anti-influenza agents (10, 30, 41–43).

What is most frustrating about viral drug resistance is that it is exceedingly challenging to predict its detailed mechanism prior to its emergence. We do recognize that gain-of-function experimentation may provide a route to predict resistance mechanisms, but the approach is not without medical risk. It is precisely here where bacteria-based assays are able to provide a solution. Conducting experiments in which the activity of the viral protein impacts bacterial phenotype enables us to harness the tremendous power of genetic screening. In other words, random mutagenesis of the viral protein followed by examining the growth rate of bacteria can readily uncover mutations that impact activity. For example, utilizing said approach enabled us to uncover all of the resistance mutations against aminoadamantanes that accumulated during five decades of use, in a matter of a few weeks (17).

The above route has now been applied to the potent drug candidate that resulted from the current study with highly encouraging results. As shown in Fig. 8, there are several mutations in the M2 channel that cause bacteria that express the particular channel to grow slower in the presence of rimantadine (e.g., S31I). This results from the fact that rimantadine blocks the M2 channel and thereby alleviates its deleterious impact on bacterial growth (Fig. 1 and ref. 16). Consequently, any mutation that causes resistance to rimantadine will slow the growth of bacteria that harbor such channels. Conversely, there are several mutations that are overrepresented in the presence of rimantadine. Such mutations presumably enhance the sensitivity of the channel to rimantadine and therefore cause the bacteria to grow faster.

Most importantly, however, is the fact that the presence of theobromine does not considerably elicit mutations that impact bacterial growth: mutations in the M2 channel can not readily change its susceptibility to be blocked by theobromine. In other words, the M2 channel is less likely to develop resistance mutations against theobromine. Moreover, the pattern of mutations that impact blockage is very different between the two drugs. For example, the mutation S31I is significantly disfavored when the bacteria are exposed to rimantadine but favored when the bacteria are exposed to theobromine. In contrast, bacteria that express a channel with the mutation I39N grow faster in the presence of rimantadine but slower in the presence of theobromine. Taken together, the virus is less likely to become resistant to the drug duo in comparison to aminoadamantanes., and the resistance pattern, were it to arrive, is distinct between the two compound classes.

## Conclusion

We present the results of a study aiming to identify new antiviral options against influenza. Our chosen route focused on a well-known drug target of the virus–its M2 H^+^ channel. Starting with a bacteria-based assay that screened a repurposed drug library, we identified a handful of potential blockers. These hits also served to search for similar compounds for further testing. Studies in tissue culture demonstrated that many of these compounds exhibit antiviral activity. Moreover, combination studies yielded a remarkable synergy between theobromine and arainosine against aminoadamantane-sensitive and aminoadamantane-resistant strains, which outperformed oseltamivir, the leading antiflu drug on the market. Importantly, the drug duo exhibited antiviral activity against two of the most important pandemic influenza strains: H1N1 swine flu and H5N1 avian flu. The tissue culture results were mirrored in an animal study that showed that the drug duo was particularly potent at lowering viral RNA and mitigating disease symptoms, far more so than oseltamivir. We recognize that developing a drug combination entails additional challenges in comparison to a single molecule. Yet, drug combinations remain an attractive approach when their activity is particular potent (e.g., Paxlovid™). Finally, fully exploiting the bacteria-based assays allowed us to demonstrate that the new drug candidate is less prone to resistance development against aminoadamantanes.

## Materials and Methods

Detailed description of the methods is provided in *SI Appendix*. In brief, the bacteria-based assays and chemical screening were conducted as previously described (16, 21–24). The in cellulo assays were conducted as described for SARS-CoV-2 (44) with the following main differences: MDCK cells were infected with the following Influenza A strains: Wisconsin/629-D02452/2009 (H1N1)pdm09, Puerto Rico/8/34(H1N1), and Israel/975/2023(H5N1). Viral RNA was quantitated with primers specific to the M gene.

### Analysis of Resistance Potential.

A library of DH10B bacteria harboring mutated M2 channels were grown with: i) no treatment, ii) 50 μM IPTG, iii) 50 μM IPTG and 100 μM rimantadine, and iv) 50 μM IPTG and 100 μM theobromine. After three hours, sequencing (Illumina; San Diego, CA, USA) was used to sequence ca. two million reads per sample.

### In Vivo Studies.

Animal experiments were conducted under IACUC-approved protocols (MD-23-17229-5 and MD-23-17159-5) and employed six-week-old male BALB/c mice. Weights and a summation of the clinical signs (*SI Appendix*, Table S1), were recorded at least twice daily. Early withdrawal and euthanasia (according to IACUC-approved protocols) occurred when a total score of 15 to 16, or 4 in the breathing rate index and/or breathing quality and/or response to stimulation and/or level of consciousness and/or more than 20% of body weight loss.

#### Tolerability.

Animals were treated by oral gavage at 10 mL/kg twice daily at different dosages up to 150 mg/kg arainosine with 100 mg/kg theobromine. The animals were given the drugs for four days, and monitoring continued 24 h after the last treatment. All drugs were dissolved in Emulphor-EL-620 and each group contained three animals.

#### Antiviral activity.

Seven groups, each containing six to eight mice were intraperitoneally anesthetized (80 mg/mL Ketamine + 10 mg/kg Xylazine IP) and intranasally infected (40 μL) at the start of day one with 10,000 viruses per animal. Subsequently, each group was treated by oral gavage (10 mL/kg) BID, except for day one in which the animals received a single half dosage. Drugs were dissolved in 1% Emulphor-EL-620 while oseltamivir was dissolved in water. During the experiment, all clinical signs and weights were recorded. After five days the animals were euthanized (according to IACUC-approved protocols) and (according to IACUC-approved protocols) and lungs were collected for further RNA quantification by RT-qPCR.

## Supplementary Material

Appendix 01 (PDF)

## Data Availability

All study data are included in the article and/or *SI Appendix*.
