# Supplementary material for: A bacteria-based search for drugs against avian and swine flu yields a potent and resistance-resilient channel blocker
Source: Proc Natl Acad Sci U S A. 2025 Aug 1;122(31):e2502240122. doi: 10.1073/pnas.2502240122 (PMC12337319; doi:10.1073/pnas.2502240122)
Supplement: Supplementary file 1 — Appendix 01 (PDF) [file pnas.2502240122.sapp.pdf]

PNAS
www.pnas.org

Supporting Information for

A Bacteria-Based Search for Drugs Against Avian and Swine Flu Yields a Potent and Resistance-Resilient Channel Blocker

Lahiri H., Israeli E., Krugliak M., Basu K., Britan-Rosich Y., Ravins Yaish T., and Arkin I.T.

Isaiah T. Arkin.
E-mail: arkin@huji.ac.il

This PDF file includes:

Supporting text
Figs. S1 to S11
Table S1
SI References

Supporting Information Text

METHODS

Bacteria-based channel assays. Three individual bacteria-based assays were used to examine the channel activity of the viroporin and blocker activity thereupon. The maltose binding protein (MBP) fusion purification system (New England BioLabs; Ipswich, MA, USA) was used, whereby influenza A (Singapore/1/1957(H2N2)) M2 was expressed as a chimera by fusing it to the carboxy-terminus of the maltose binding protein to ensure proper membrane reconstitution (1). The aminoadamantane-resistance mutation, S31N (2), was obtained by site-directed mutagenesis of the above Singapore strain, which is aminoadamantane-sensitive (3). Sequences of these and additional M2 proteins are given in supporting Fig. 1.

Negative assay. DH10B cells (Invitrogen; Carlsbad, CA, USA) containing influenza-M2 and S31N-M2 plasmids were grown overnight in lysogeny broth (LB) medium. Following a 50-fold dilution, the culture was set to grow until its OD_{600} reached a value of 0.2. Subsequently, varying concentrations of Isopropyl-β-D-1-thiogalactopyranoside (IPTG) ranging from 25 µM to 50 µM at 5 µM intervals were employed to evaluate the impact of protein expression on bacterial growth. Bacteria grown in 96-well plates were incubated in a multiplate reader (LogPhase 600 from BioTek; Santa Clara, CA, USA) at 37°C for 16 hrs, and readings were taken at 15 min intervals.

Positive assay. K^+-uptake-deficient bacteria (LB650: *ΔtrkG*, *ΔtrkH*, and *ΔkdpABC5*) were used in this assay. These particular bacteria are incapable of growth in regular LB medium, unless they express a channel capable of potassium transport, or if the growth medium contains a high concentration of potassium (4). Overnight growth of bacteria was conducted in LBK medium, which was identical to LB expect that KCl replaced NaCl. Different concentrations of KCl and Isopropyl-β-D-1-thiogalactopyranoside were used to gauge the impact of the channel's activity on the bacterial phenotype.

pHlux assay. The pHlux assay (5) is based on LR1 bacteria that express a chromosomal copy of a pH-sensitive GFP (6). The bacteria were grown overnight in the same conditions as DH10B cells. Subsequently, the culture it is diluted to 1:50 and again grown until the OD_{600} reaches a values of 0.2. Different concentrations of Isopropyl-β-D-1-thiogalactopyranoside are used to induce protein expression for one hour and cells are diluted to an OD_{600} of 0.3 and pelleted down at 3500 g for 10 min. The cells are then washed twice and resuspended in McIlvaine Buffer(7) which contains 200 mM Na_2HPO_4 and 0.9% NaCl adjusted to pH 7.6 with 0.1 M citric acid. The spectroscopy is undertaken in 96-well plates (Nunclon f96 Microwell Black Polystyrene, Thermo Fisher Scientific; Waltham, MA, USA), and in each well 200 µL of cell suspension was added along with 30 µL of buffer. 70 µL of citric acid (300 mM, 0.9% NaCl) are then added to each well by a liquid handling system (Tecan; Männedorf, Switzerland) and readings were carried out at two excitations of 390 nm and 466 nm with emission held at 520 nm (6). The proton flow was calculated from the ratio of two excitations as described previously (5).

Channel blocker screening. A library of 2,839 compounds was purchased from MedChem Express (HY-L035, Monmouth Junction, NJ, USA) and used for screening against the aminoadamantane-sensitive and S31N (aminoadamantane-resistant) influenza virus M2 channels. The screening employed the negative assay with a 100 µM drug concentration with a final concentration of DMSO of 2%. Protein expression was achieved by adding 45 µM of Isopropyl-β-D-1-thiogalactopyranoside which retarded growth by approximately 50% (Fig. S2). Bacteria that received only DMSO were designated as negative control. Two metrics were recorded: growth rate and final growth density. Drugs that were able to enhance bacterial growth by a certain threshold were subsequently checked in duplicate.

Successful compounds were also subjected to the positive genetic assay where they were examined at 10 µM Isopropyl-β-D-1-thiogalactopyranoside and 8 mM KCL conditions. Finally, compounds that passed both the negative and positive assays were subjected to the pHlux assay.

Analysis of resistance potential. The plasmid containing the aminoadamantanes-sensitive M2 protein was mutated with the Genemorph II Random Mutagenesis Kit (Stratagene; California, La Jolla, CA, USA) focusing on the transmembrane region of the protein (Ser22–Leu43). Gibson assembly (New England BioLabs) was subsequently used to insert the resulting PCR products in the pMAL-p2X vector (New England BioLabs).

Subsequently, DH10B bacteria were transformed with the plasmid library and the following four different treatments were conducted: (i) LB media, (ii) LB media and 50 µM Isopropyl-β-D-1-thiogalactopyranoside, (iii) LB media, 50 µM Isopropyl-β-D-1-thiogalactopyranoside and 100 µM rimantadine, and (iv) LB media, 50 µM Isopropyl-β-D-1-thiogalactopyranoside and 100 µM theobromine. The bacteria were grown for three hours in the selected media and next-generation sequencing employing Illumina sequencing by synthesis technology (San Diego, CA, USA) was used to sequence the library. Approximately two million reads were obtained for each sample, and analyses were conducted for reads containing 50% or higher identity to the wild type sequence.

In-vitro studies.

Cell culture. Madin-Darby canine kidney (ATCC MDCK NBL-2) cells were maintained in Dulbecco's Modified Eagle's Medium (DMEM) (Biological Industries; Beit Haemek, Israel), supplemented with 10% fetal bovine serum, 2 mM L-Glutamine, 10 IU/mL Penicillin, 10 µg/mL streptomycin, and Biomycin-3 (Biological Industries).

Virus culture and infection. Influenza A Virus A/Wisconsin/629-D02452/2009 (H1N1)pdm09 was obtained through BEI Resources, NIAID, NIH (NR-19810). Influenza A Virus A/Puerto Rico/8/34 was acquired from ATCC (VR-1469). Influenza H5N1, A/Israel/975/2023 was collected from a peregrine falcon and obtained from the Israeli veterinary service. The M2 sequences from the three viruses are given in Fig. S1. Identical aliquots of virus stock were prepared from the mother stock. For infection, 1:1000 diluted sub-stocks were prepared from each aliquot. Subsequent infection of MDCK cells was carried out in DMEM containing 0.3% Bovine Serum Albumin, (Sigma Aldrich) and 3 µg/ml TPCK treated trypsin (Sigma Aldrich) and further incubated for 48 hrs at 37°C in a 5% CO_2 atmosphere. All infection experiments were performed in a BSL-3 facility (Barry Skolnick National Biosafety Level 3 biocontainment unit at the Hebrew University of Jerusalem, Israel).

Cell viability and cytotoxicity assays. MDCK cells were cultured in a 96-well plate with 200 µL of medium at density of 15,000 cells per well and grown overnight. The dilutions of tested compounds were prepared in DMEM with 0.3% BSA, 3 µg/ml TPCK-treated trypsin, and 50 µL of said solution was added to the cells. The effects of the drugs on the viability of the cells were assessed at 48 hrs post-treatment using CellTiter 96 Aqueous Non-Radioactive Cell Proliferation reagent (Promega; Madison, WI, USA). To examine the effect of different drugs, cells were infected with the influenza virus at a multiplicity of infection (MOI) of 0.3 for two hours followed by treatment with various drugs. Infection with the PR8 strain employed 3 ug/ml of TPCK treated trypsin and an MOI of 1. Each compound concentration was tested in triplicate and each assay plate contained the following controls: no cells (background control), cells treated with medium (mock infection for normalization), infected/untreated cells, and infected/solvent-treated cells (infection control).

At two days post-infection, anti-viral drug efficacies were assessed by the CellTiter 96 Aqueous Non-Radioactive Cell Proliferation reagent (Promega) for 3 hrs at 37°C in a 5% CO_2 atmosphere. Reactions were stopped and the virus was inactivated by adding 30 µL of 4% formaldehyde. Absorbance was measured at 492 nm using a Tecan plate reader Finally, the data were normalized to the mock-infected control, after which inhibition metrics values were calculated by fitting the data to a Monod equation (half-saturation coefficient or K_s).

Viral load quantification. To check viral load from each experiment, the supernatant was collected after 48 hrs of infection and RT-qPCR was performed. Viral RNA was extracted from the supernatant using the Aurum™ total RNA mini-Kit (Bio-Rad; Hercules, CA, USA). Subsequently, cDNA was synthesized from the extracted viral RNA according to the qScript cDNA synthesis kit protocol (Quanta bio; Beverly, MA, USA).

Detection of influenza virus RNA was done with primers specific to the M gene: forward primer 5'-CGCTCAGACATGAG-AACAGAATGG-3' and reverse primer 5'-TAACTAGCCTGACTAGCAACCTC-3' (Integrated DNA Technologies; Coralville, IA, USA). The reaction was conducted in a 96-well plate, with 2 µL of cDNA, 1× iTaq Universal SYBR green supermix (Bio-Rad) and 200 nM forward and reverse detection primers to a total volume of 20 µL/well.

For a standard curve from purified RNA, a stock concentration of 10^6 copies/ml was used and cDNA was prepared and then serially diluted with elution buffer. Thermal cycling was performed using StepOnePlus™ Real-Time PCR System with StepOne™ Software Version 2.3 (Carlsbad, CA, USA).

Structural similarity studies. Chemicals that are structurally similar to the hit compounds (obtained from the initial channel screening and *in-vitro* studies) were searched to examine their anti-viral activity. In this regard, Tanimoto similarity searches with a value of 85% and above were performed with each hit compound to get several similar chemicals available commercially.

***In-vivo* studies.** All procedures involving animals took place at the Authority for Biological and Biomedical Models of the Hebrew University of Jerusalem, accredited by the Israeli Council for Experiments of Animal Subjects and by the Association for Assessment and Accreditation of Laboratory Animal Care, International (AAALAC). The anti-viral efficacy experiments were conducted at the Barry Skolnick national biosafety Level 3 biocontainment unit. Finally, all experiments were conducted under IACUC-approved protocols (MD-23-17229-5 and MD-23-17159-5).

All animal experiments employed six-week-old male BALB/c mice. Throughout the experiment, a scoring table was used to monitor the condition of the animals alongside recording body weights daily. Death is not an endpoint in the experiment. Rather, in case of more than 20% of body weight loss, euthanasia was performed. In addition, a summation of the clinical signs according to the General Mouse Scoring Table (see Table S1), was recorded at least twice daily. The animals were monitored thrice a day if the score is higher than 12. An early withdrawal point and euthanasia occured when a total score of 15-16 or 4 in the breathing rate index and/or breathing quality and/or response to stimulation and/or level of consciousness will be observed.

Tolerability. The tolerability of test articles was assessed in healthy mice. Animals were treated by oral gavage (see below) at 10 mL/kg (ca. volume of 0.2 mL) twice daily (eight hours apart) at the different dosages (which represents the total daily dosage). The animals were be given the drugs for four days, and monitoring was continued 24 hours after the last treatment.

The following dosages were employed representing a 1:1 drug combinations: (i) 1.5 mg/kg arainosine and 1 mg/kg theobromine, (ii) 4.5 mg/kg arainosine and 3 mg/kg theobromine, (iii) 15 mg/kg arainosine and 10 mg/kg theobromine, (iv) 45 mg/kg arainosine and 30 mg/kg theobromine and, (v) 150 mg/kg arainosine and 100 mg/kg theobromine. All drugs were dissolved in Emulphor-EL-620. Each group contained three animals.

Anti-viral activity. Anti-viral efficacy studies were conducted on mice which were divided into seven groups, each containing six to eight mice. Each group of mice was intraperitoneally anesthetized (80 mg/mL Ketamine + 10 mg/kg Xylazine IP) and infected

at the start of day one with 10,000 viruses per animal by intranasal administration of 40 µL. The inoculum size was determined as one that induced appreciable disease symptoms without reaching lethality. Subsequently, each group was treated by oral gavage (10 mL/kg) BID, according to the treatment specified below, except for day one in which the animals received only a single treatment of half a daily dosage.

The seven groups were treated as follows: (i) 1% Emulphor-EL-620 (vehicle control), (ii) oseltamivir 20 mg/kg, (iii) 1.5 mg/kg arainosine and 1 mg/kg theobromine, (iv) 4.5 mg/kg arainosine and 3 mg/kg theobromine, (v) 15 mg/kg arainosine and 10 mg/kg theobromine, (vi) 45 mg/kg arainosine and 30 mg/kg theobromine and, (vii) 150 mg/kg arainosine and 100 mg/kg theobromine. All the combined drugs in desired concentrations were dissolved in an aqueous solution of 1% Emulphor-EL-620 while oseltamivir was dissolved in water.

The experiment's duration was five days during which all clinical signs were recorded including weight. After five days the animals were sacrificed and lungs were collected for further RNA quantification by RT-qPCR.

Chemicals. Isopropyl-β-D-1-thiogalactopyranoside was purchased from Biochemika-Fluka (Buchs; Switzerland). Xanthine and 3-methyl xanthine were purchased from Acros Organics (Antwerpen, Belgium). 1-methyl xanthine and paraxanthine were purchased from ChemScene (Monmouth Junction, NJ, USA). Arainosine was purchased from BOC Sciences (Shirley, NY, USA). Enprofylline and 7-methyl xanthine were purchased from Glentham Life Sciences (Corsham, United Kingdom) and Alfa Aesar (Ward Hill, MA, US), respectively. All other chemicals were purchased from Sigma-Aldrich laboratories.

Supporting figures

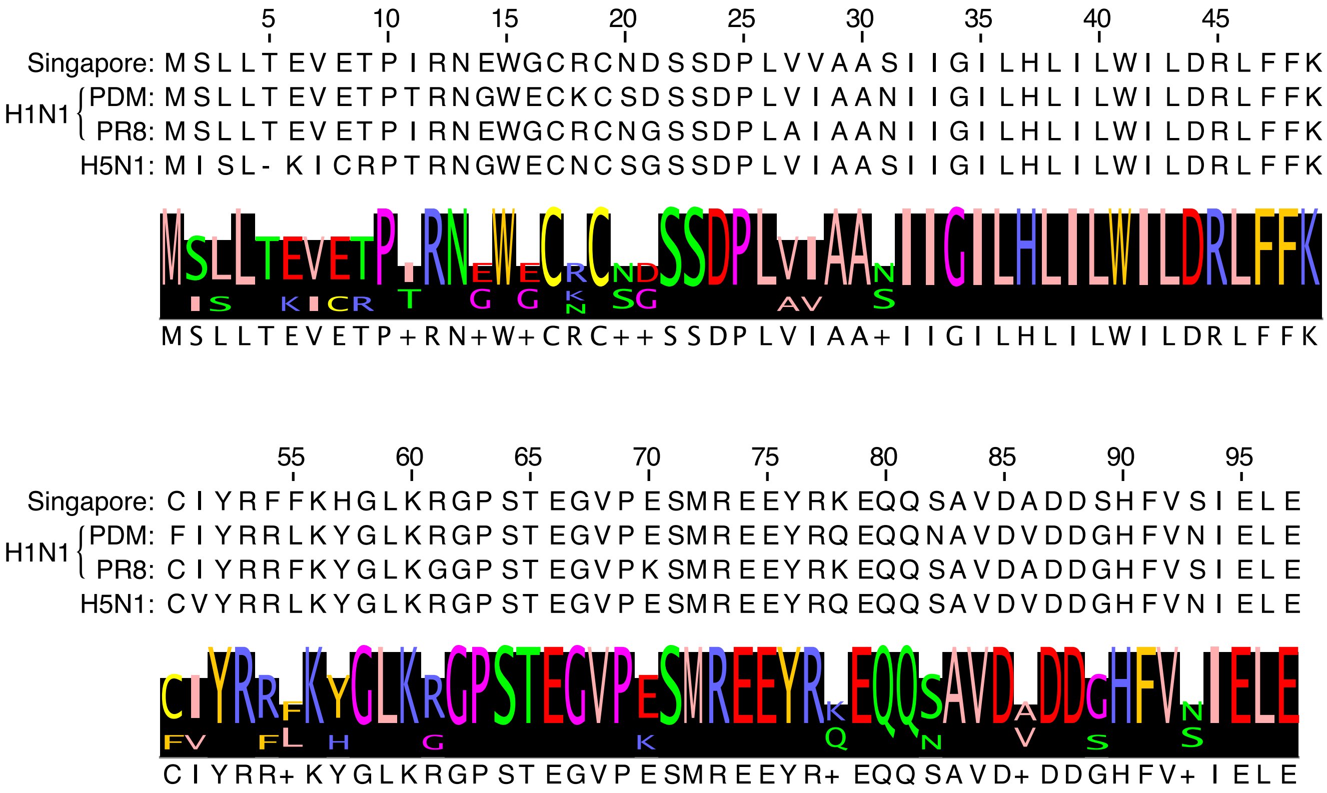

Fig. S1. Sequences of the different influenza virus M2 proteins used in the study. The sequence of the Singapore strain (A/Singapore/1/1957(H2N2)) was used in the bacteria-based screening, while the bottom three represent the M2 protein from the viruses used in the *in cellulo* and *in vivo* studies. The PDM strain is: A/Wisconsin/629-D02452/2009; the PR8 strain is: A/Puerto Rico/8/34; and the H5N1 strain is: A/Israel/975/2023

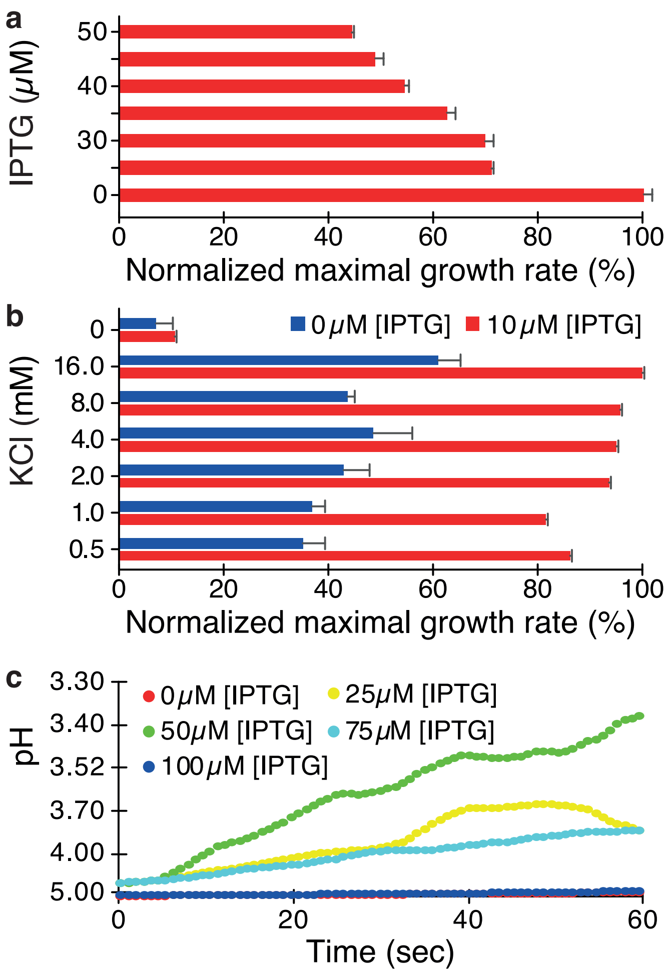

Fig. S2. Three bacteria-based assays to assess the channel activity of the influenza M2 protein: (a) Negative assay, (b) Positive assay, and (c) pH-dependent fluorescence (pHlux) assay. Note that in the negative assay, protein expression retards bacterial growth; in the positive assay, protein expression increases growth; and finally, in the fluorescence-based essay, protein expression changes bacterial pH.

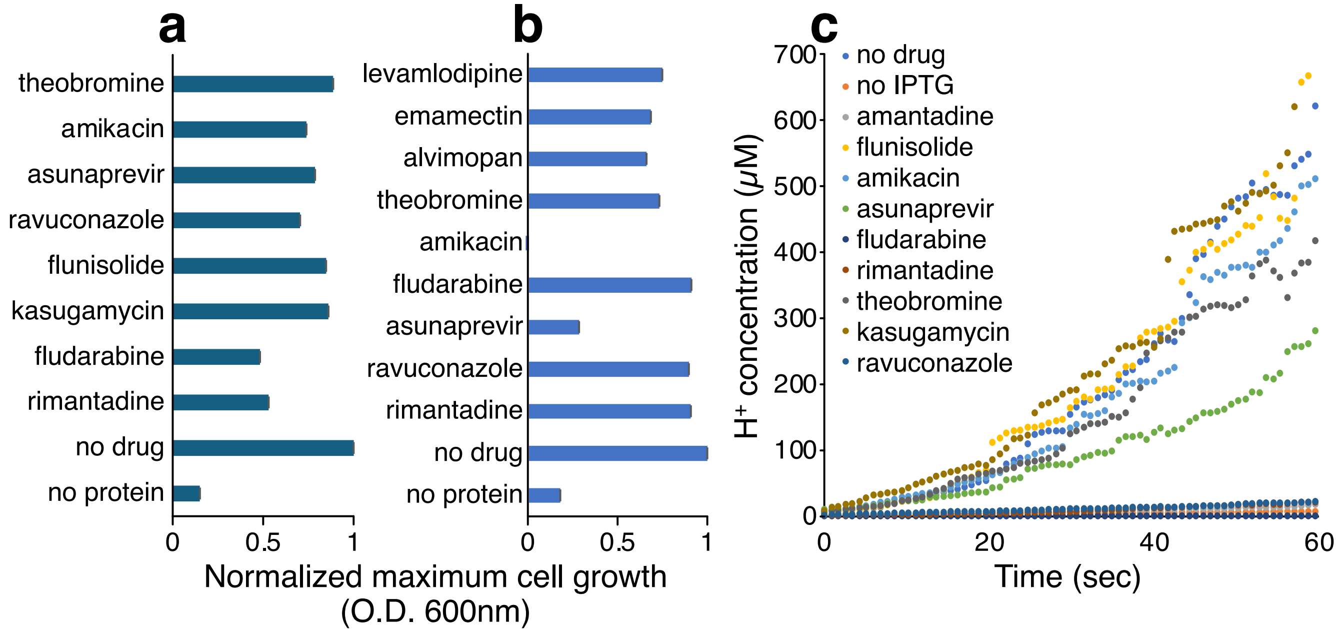

Fig. S3. Bacteria-based positive assay to assess the activity of screened drugs against the aminoadamantane-sensitive M2 channel (a) and against a channel containing the S31N mutation (b). Panel c depicts the pH-dependent fluorescence assay on an aminoadamantane-sensitive M2 channel and the effects of drugs thereupon.

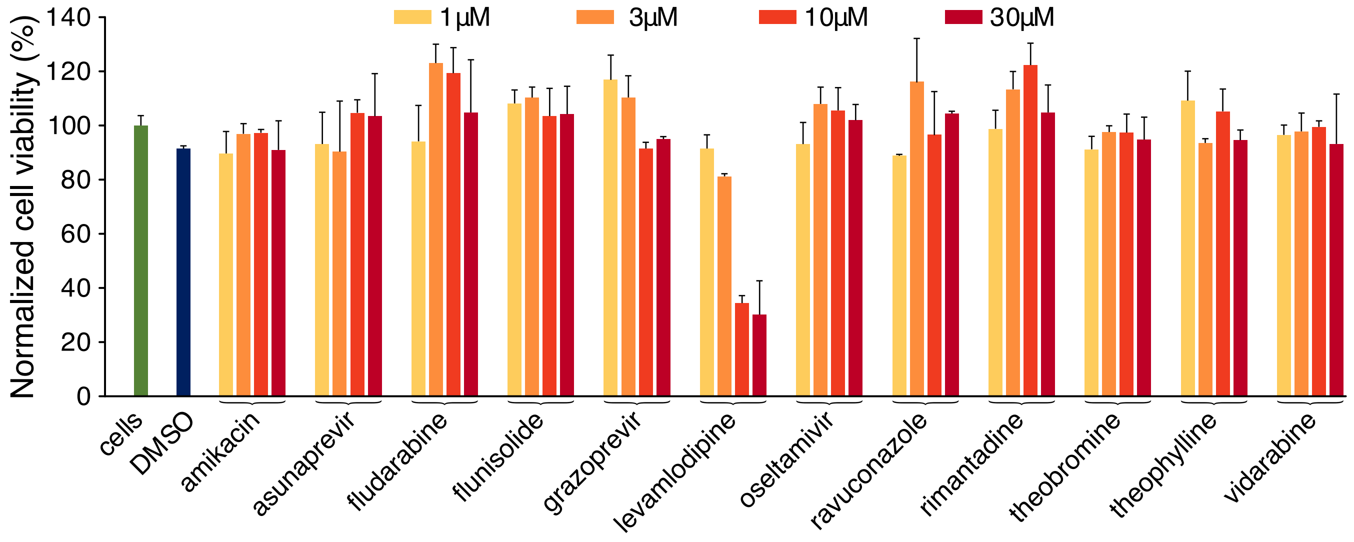

Fig. S4. Toxicity of different compounds in tissue culture. Cellular viability after 48 hours was monitored by MTS as a function of different compounds at four concentrations, as noted. Untreated cells were used as a comparison and normalized to 100%. DMSO represents the vehicle.

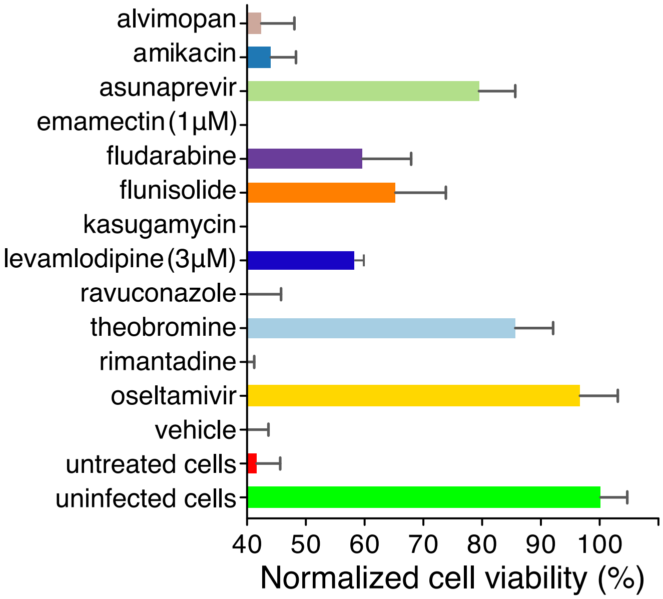

Fig. S5. Anti-viral activity of M2 channel blockers. MDCK cells were infected with an H1N1 virus at an MOI of 0.3 and their viability was monitored by MTS after 48 hrs. Effects of different drugs at a concentration of 10 µM in 0.1% DMSO are listed. Emamectin and Levamlodipine were tested at 1 and 3 µM concentration, respectively, due to their toxicity. Results are normalized relative to uninfected cells.

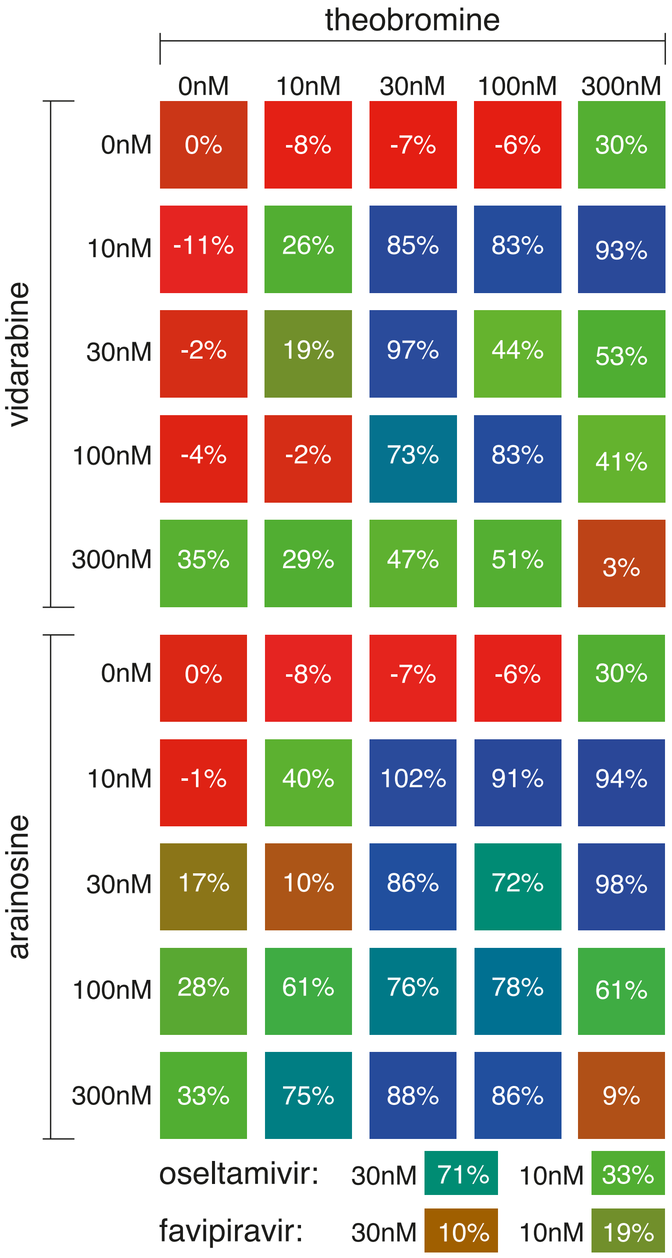

Fig. S6. Synergy analyses of anti-viral *in cellulo* activity between theobromine and vidarabine and between theobromine and arainosine. The anti-viral activity of two approved drugs at 30 nM and 10 nM are shown at the bottom for comparison. MDCK cells were infected with the H1N1 influenza A virus at an MOI of 0.3 and their viability was monitored by MTS after 48 hrs. Results are normalized relative to uninfected cells and untreated cells and represent the average of at least three measurements.

	no nucleoside	fludarabine	vidarabine	nelarabine	adenosine	cordycepin	clofarabine	cladribine	arainosine
no xanthine	0%	11%	-21%	-22%	-31%	-21%	-32%	-29%	-23%
theobromine	-24%	16%	61%	-17%	-23%	-15%	-15%	-10%	70%
caffeine	-29%	-22%	-25%	-13%	3%	7%	-24%	-6%	-6%
theophylline	6%	-9%	-6%	-18%	-6%	-11%	0%	-3%	-25%
enprophylline	-22%	-10%	-12%	6%	7%	-10%	-4%	5%	-8%
paraxanthine	-32%	-22%	-11%	-24%	-7%	-18%	-15%	-17%	-18%
7-methylxanthine	-27%	-12%	5%	0%	-7%	4%	-15%	-29%	-7%
xanthine	-29%	-20%	-4%	-4%	0%	-20%	-5%	2%	-20%
1-methylxanthine	-24%	-3%	7%	-2%	-4%	3%	-17%	-16%	-1%
3-methylxanthine	-31%	-15%	15%	-6%	-14%	-18%	-5%	2%	2%

Fig. S7. Combination anti-viral *in cellulo* studies of nucleoside analogs and xanthine analogs. MDCK cells were infected with the H1N1 virus at an MOI of 0.3 and their viability was monitored by MTS after 48 hrs. Results are normalized relative to uninfected cells and untreated cells and represent the average of at least three measurements. All compounds were at at 100 nM.

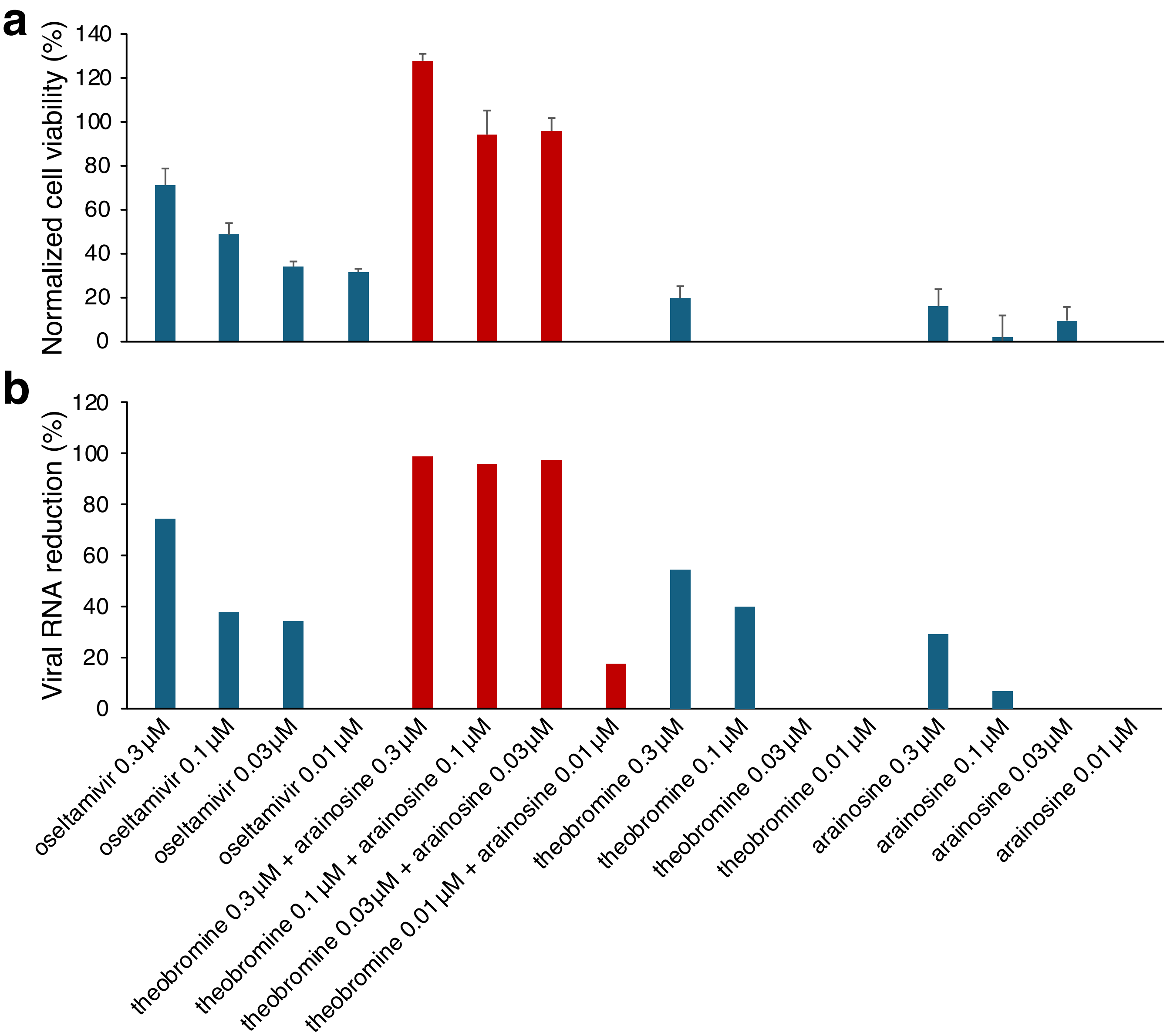

Fig. S8. Antiviral activity (a) and viral load reduction (b) for different combinations of theobromine and arainosine on PR8 H1N1 influenza strain.

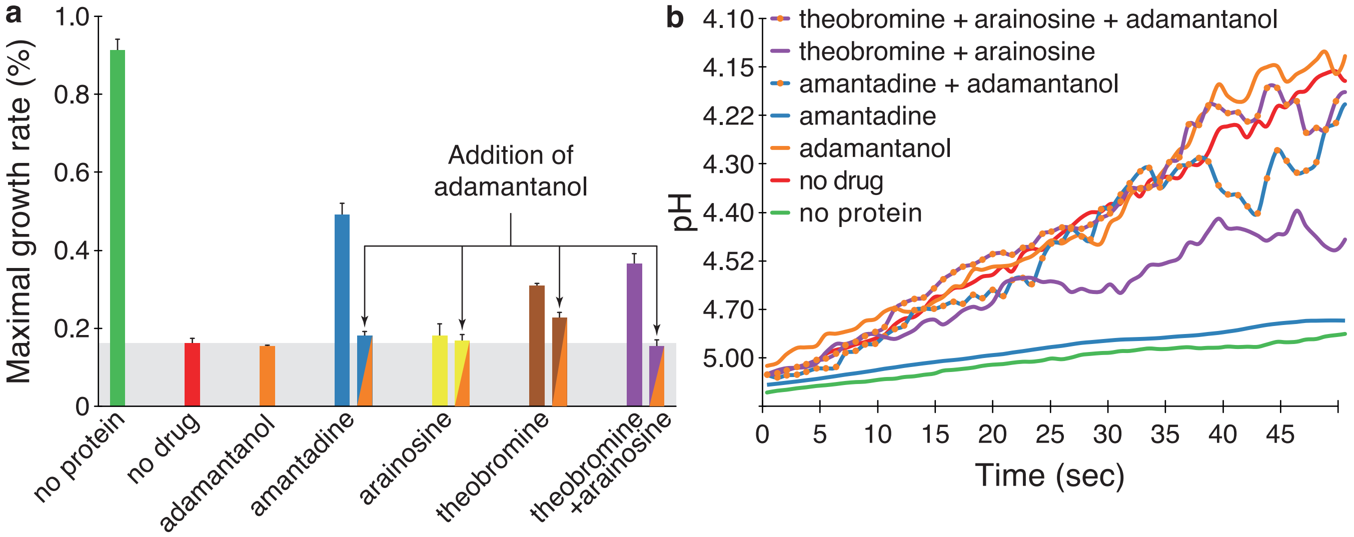

Fig. S9. Effect of adamantanol on the activities of amantadine, theobromine, arainosine, and the theobromine-arainosine combination in the bacteria-based assays. (a) Negative assay in which bacterial growth is retarded by the M2 channel, and consequently blockers increase growth rate. (b) Fluorescence based assay, in which the M2 protein increases acidification, but effective blockers neutralize the channel's activity. Note that adamantanol reduces the activity of the drugs in both assays, except for arainosine that exhibits a minor reduction.

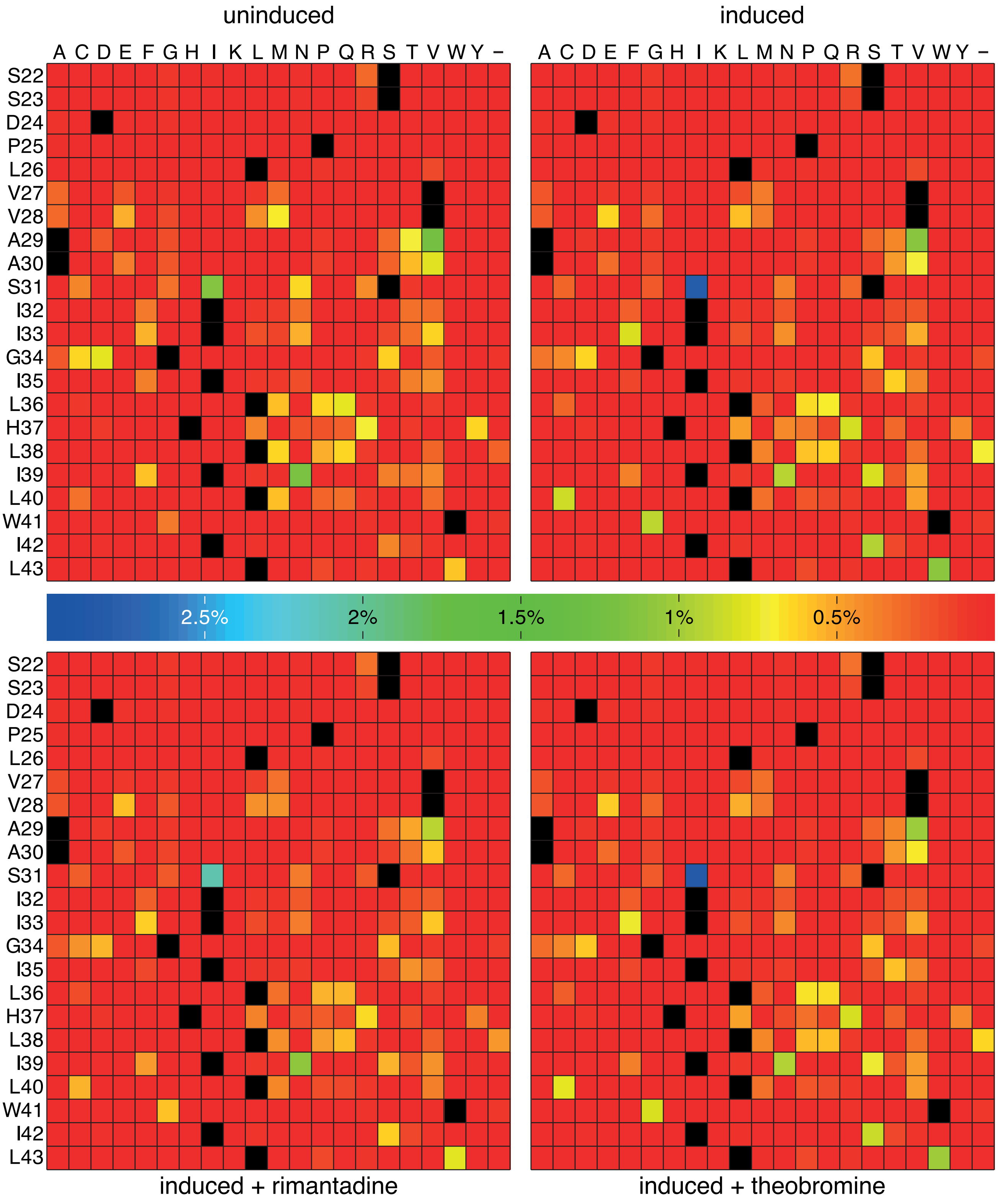

Fig. S10. Deep sequencing results of an analysis to exhaustively map the resistance potential of the M2 channel against blockers. A library of bacteria harboring a randomly mutated M2 channel was grown in liquid media for three hours after which their plasmids were sequenced. The prevalence of every amino acid is tabulated and color-coded, whereby black represents the wild type. The four different treatments are indicated in the figure.

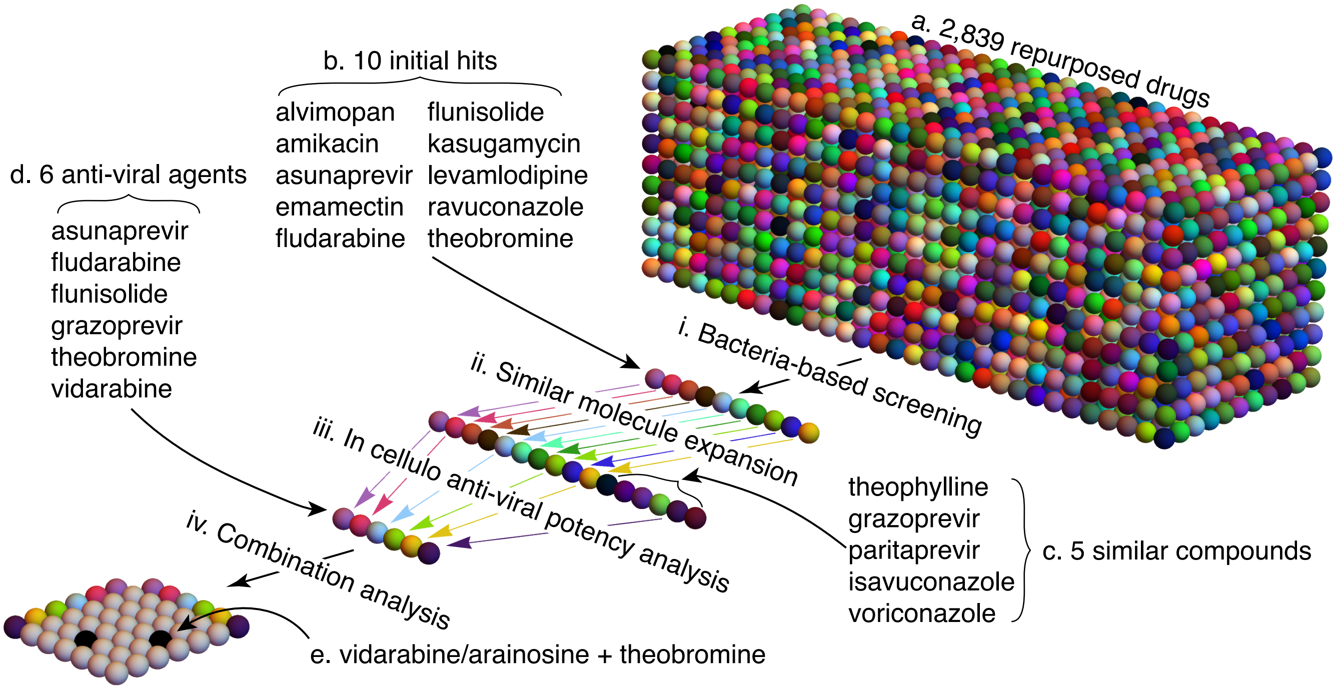

Fig. S11. Systematic approach for anti-influenza agents yielding the theobromine and arainosine drug duo: i. A repurposed drug library of 2,839 compounds (a) was screened with bacteria-based assays to yield ten hits (b). ii. Five additional compounds (c) were found based on their similarity to the ten hits from the previous stage (b). iii. *In cellulo* testing demonstrated that six compounds (d) exhibit anti-viral activity out of the 15 expanded hits (b+c). iv. One pair of compounds exhibits remarkable anti-viral synergism from combination studies in tissue culture. Note that two combinations are shown due to the matrix's symmetry.

Variable	Score	Description
Appearance	0	Coat is smooth
	1	Patches of hair piloerected
	2	Majority of back is piloerected
	3	Piloerection may or may not be present, hunched back
	4	Piloerection may or may not be present, mouse appears emaciated
Level of consciousness	0	Mouse is active
	1	Mouse is active but avoids standing upright
	2	Mouse activity is noticeably slowed. The mouse is still ambulant.
	3	Activity is impaired. Mouse only moves when provoked, movement have a tremor
	4	Activity severely impaired. Mouse remains stationary when provoked, with possible tremor
Activity	0	Normal amount of activity. Mouse is any of: eating, drinking, climbing, running, fighting
	1	Slightly suppressed activity. Mouse is moving around bottom of cage
	2	Suppressed activity. Mouse is stationary with occasional investigative movements
	3	No activity. Mouse is stationary
	4	No activity. Mouse experiencing tremors, particularly in the hind legs
Response to stimulus	0	Mouse response immediately to auditory stimulus or touch
	1	Slow or no response to auditory stimulus; strong response to touch (moves to escape)
	2	No response to auditory stimulus; moderate response to touch (moves a few steps)
	3	No response to auditory stimulus; mild response to touch (no locomotion)
	4	No response to auditory stimulus; little or no response to touch. Cannot right itself if pushed over
Eyes	0	Open
	1	Eyes not fully open, possibly with secretions
	2	Eyes at least half closed, possibly with secretions
	3	Eyes half closed or more, possibly with secretions
	4	Eyes closed or milky
Respiratory rate	0	Normal, rapid mouse respiration
	1	Slightly decreased respiration (rate not quantifiable by eye)
	2	Moderately reduced respiration (rate at the upper range of quantifying by eye)
	3	Severely reduced respiration (rate easily countable by eye, 0.5s between breaths)
	4	Extremely reduces respiration (>1s between breaths)
Respiration quality	0	Normal
	1	Brief periods of labored breathing
	2	labored, no gasping
	3	labored with intermittent gasps
	4	Gasping

Table S1. General mouse scoring table.

References

1. Assa D, Alhadeff R, Krugliak M, Arkin IT (2016) Mapping the resistance potential of influenza's h+ channel against an antiviral blocker. *Journal of molecular biology* 428(20):4209–4217.
2. Hay A, Wolstenholme A, Skehel J, Smith MH (1985) The molecular basis of the specific anti-influenza action of amantadine. *The EMBO journal* 4(11):3021–3024.
3. Astrahan P, Kass I, Cooper MA, Arkin IT (2004) A novel method of resistance for influenza against a channel-blocking antiviral drug. *Proteins* 55(2):251–7.
4. Stumpe S, Bakker EP (1997) Requirement of a large K^+-uptake capacity and of extracytoplasmic protease activity for protamine resistance of escherichia coli. *Archives of microbiology* 167:126–136.
5. Santner P, et al. (2018) A robust proton flux (phlux) assay for studying the function and inhibition of the influenza a m2 proton channel. *Biochemistry* 57(41):5949–5956.
6. Miesenböck G, De Angelis DA, Rothman JE (1998) Visualizing secretion and synaptic transmission with ph-sensitive green fluorescent proteins. *Nature* 394(6689):192–195.
7. McIlvaine T (1921) A buffer solution for colorimetric comparison. *J. biol. Chem* 49(1):183–186.
